# Development of Potent Type V MAPK Inhibitors: Design, Synthesis, and Biological Evaluation of Benzothiazole Derivatives Targeting p38α MAPK in Breast Cancer Cells

**DOI:** 10.1002/ardp.202500011

**Published:** 2025-04-07

**Authors:** Bayan Zoatier, K. Gizem Yildiztekin, M. Abdullah Alagoz, Ceylan Hepokur, Serdar Burmaoglu, Oztekin Algul

**Affiliations:** ^1^ Department of Pharmaceutical Chemistry, Faculty of Pharmacy Mersin University Mersin Türkiye; ^2^ Department of Toxicology, Faculty of Pharmacy Erzincan Binali Yıldırım University Erzincan Türkiye; ^3^ Department of Pharmaceutical Chemistry, Faculty of Pharmacy Inonu University Malatya Türkiye; ^4^ Department of Biochemistry, Faculty of Pharmacy Sivas Cumhuriyet University Sivas Türkiye; ^5^ Department of Chemistry, Faculty of Science Atatürk University Erzurum Türkiye; ^6^ Department of Pharmaceutical Chemistry, Faculty of Pharmacy Erzincan Binali Yıldırım University Erzincan Türkiye

**Keywords:** antiproliferative effect, benzothiazole, MCF7, molecular docking, MTT assay, p38α MAPK

## Abstract

Type V MAPK inhibitors are distinguished by their capacity to target both the ATP binding site and a specific allosteric site on the enzyme. The present work utilized in silico analysis with Maestro 13.8.135 (Schrodinger) software in conjunction with experimental investigations to enhance the antiproliferative efficacy and forecast the likely mechanism of action of benzothiazole derivatives. Approximately 28 compounds were developed, produced, and assessed for their antiproliferative properties against two breast cancer cell lines: ER+ (MCF7) and ER‐ (MDA‐MB‐231), in addition to one normal mouse fibroblast cell line (L929). Their antiproliferative activities were evaluated via the MTT test, with doxorubicin and cisplatin serving as reference drugs for comparison. Consequently, the compounds with the greatest activity against the MCF7 cell line were chosen, and their inhibitory effects on the p38α MAPK enzyme were examined. The molecular docking studies of compounds **15** and **19** demonstrated significant binding affinities for p38α MAPK. Molecular dynamics simulations conducted over 100 ns revealed that compounds **15** and **19** exhibit stability inside both the ATP‐binding domain and the lipid domain of p38α MAPK. The research focused on creating effective Type V MAPK inhibitors demonstrate that compounds **15** and **19** possess considerable ability to inhibit p38α MAPK, hence establishing them as promising anticancer agents.

## Graphical abstract

Type V MAPK inhibitors are unique for targeting both ATP and allosteric sites. In silico analysis and experimental methods were combined to optimize benzothiazole derivatives for antiproliferative activity against breast cancer cells. Two compounds showed significant inhibition of p38α MAPK, suggesting promising anticancer potential.

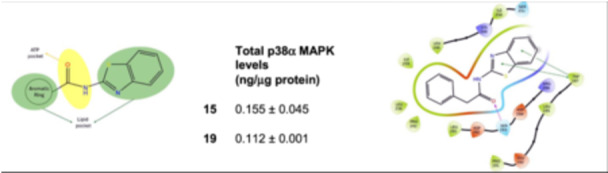

## 1 Introduction

Mitogen‐activated protein kinase (MAPK) cascades are signaling pathways that participate in various cellular activities, such as proliferation, differentiation, and growth [1]. The MAPK signaling cascade has three principal routes: (a) ERK1/2, which is activated by mitogens; (b) Jun N‐terminal kinase (JNK); and (c) p38 pathways, which are stimulated by stress and genotoxic factors. The p38 kinase, a multifunctional enzyme, governs numerous cellular activities [2]. Among the MAPK isoforms, p38 MAPK is recognized as one of the most thoroughly investigated. p38 MAPK modulates diverse biological functions, such as cell proliferation, differentiation, apoptosis, and inflammation, by phosphorylating serine and/or threonine residues of target proteins [3]. Four isoforms of p38 MAPKs exist: p38α, β, γ, and δ, each demonstrating distinct regulatory mechanisms and activities, especially regarding cell death pathways, which vary based on the cellular context, stimuli, and isoform expression patterns [4, 5, 6]. Among the four isoforms, p38α is the most thoroughly described and is expressed universally across all organs. Dysregulation of this factor has been linked to carcinogenesis, especially in breast cancer, rendering it a potential therapeutic target. Elevated p38α levels are significantly associated with poor prognosis and highly aggressive breast cancer [7, 8, 9, 10]. Selective activation of p38α is observed in grade II or III intraductal breast cancers, but its downregulation induces antitumor responses [9, 11, 12]. Thus, p38α inhibition has surfaced as a prospective approach for the treatment of breast cancer, especially in ER‐negative variants [13].

**Figure 1:**
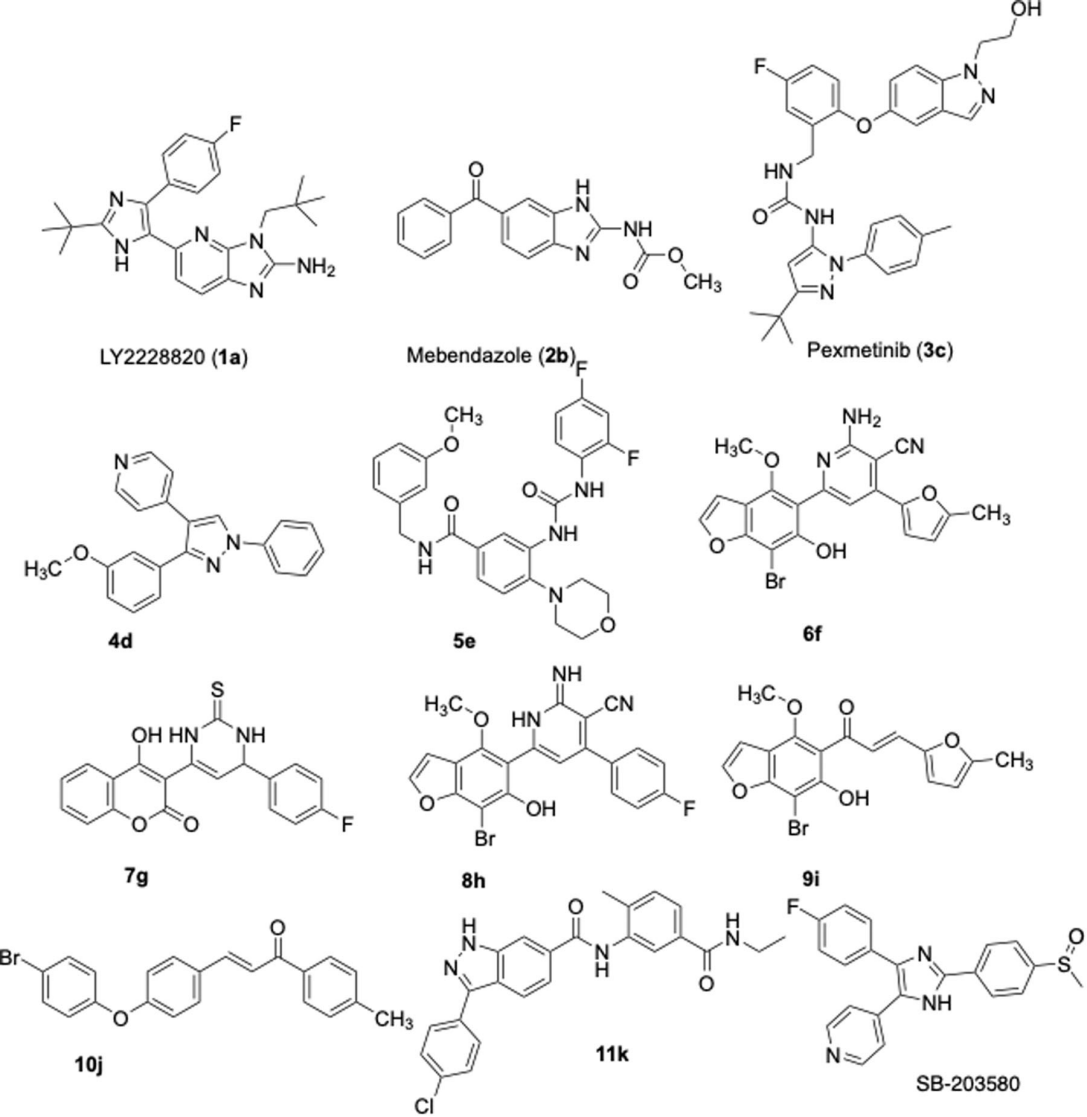
Previously reported inhibitors of MAPK with anticancer/antiproliferative activity in the literature.

Due to the significance of p38α MAPK in several physiological and pathological processes, numerous inhibitors have been identified throughout the years. Despite significant endeavors to create inhibitors for p38α MAPK [14, 15, 16, 17], none have attained FDA approval, mostly due to off‐target effects and restricted in vivo efficacy [18]. Significant compounds, illustrated in Figure 1, including LY2228820 (**1a**) [19], mebendazole (**2b**) [20], and pexmetinib (**3c**) [21], have demonstrated potential in preclinical and clinical investigations; nonetheless, obstacles remain in attaining selectivity and efficacy. Compound **4d** exhibits relative selectivity for p38α (IC_50_ = 515 nM) in comparison to 15 other kinases [22]. Compound **5e** inhibited the viability and reduced the proliferation and migration of prostate cancer cells without causing considerable damage to normal prostate cells [23]. The nicotinonitrile‐benzofuran hybrid (**6f**) demonstrated substantial action as a p38α MAP kinase inhibitor, with an IC_50_ of 0.040 μM [24]. Thioxopyrimidine derivative **7g** suppressed p38α MAPK activation in HeLa cells by 0.64 compared with the control anisomycin (30 μM), resulting in significant reductions in cell viability (80% and 72%) at concentrations of 10 and 30 μM, respectively [25]. Fluorophenyl‐2‐iminopyridine‐benzofuran (**8h**) had a promising efficacy against p38α MAPK, exhibiting an IC_50_ value of 0.27 μM, superior to the reference compound SB203580, which has an IC_50_ value of 0.50 μM [26]. The bromobenzofuryl chalcone derivative (**9i**) exhibited remarkable antiproliferative activity in the MTT assay, with IC_50_ values of 1.35 and 2.09 μM for the MCF7 and MDA‐MB‐231 cell lines, respectively, in comparison to lapatinib, which had IC_50_ values of 4.69 μM for both cell lines. The in vitro study showed that compound **9i** (IC_50_ value of 0.04 μM) inhibited p38α MAP kinase more effectively than the reference drug SB203580 (IC_50_ value of 0.50 μM) [24]. The phenoxy chalcone derivative (**10j**) reduced both total p38α MAPK and phosphorylated enzyme levels in MCF‐7 cells [27]. Recently, 3‐(4‐chlorophenyl)‐N‐(5‐(ethylcarbamoyl)‐2‐methylphenyl)‐1*H*‐indazole‐6‐carboxamide, (**11k**) was characterized with an IC_50_ value of 3.37 ± 0.24 μM against p38α MAPK [28].

Due to the pivotal function of p38α MAPK in cellular stress responses and its link to cancer, current endeavors are aimed at identifying new inhibitors. These inhibitors, classified as ATP‐site binders and non‐ATP‐site binders, seek to address current limitations and enhance treatment efficacy. Inhibitors have been engineered to target many locations on p38α MAPK. p38α MAPK comprises two lobes: the C‐terminal lobe and the N‐terminal lobe. Substrate contact occurs in the C‐terminal lobe. The N‐terminal lobe contains an ATP‐binding site and a catalytic site. Numerous research have produced inhibitors aimed at the ATP‐binding region of MAPK; however, the anticipated effectiveness was not realized due to issues of selectivity and toxicity. Researchers are undertaking investigations to develop selective inhibitors that precisely target distinct regions of MAPK [29, 30]. MAPK inhibitors are categorized into six classes, Types I–VI, based on their modes of inhibition. The predominant strategy for MAPK inhibition involves Types I and II inhibitors, designed to obstruct ATP binding at the ATP binding site of MAPK in both its active and inactive states, respectively [31]. Thirty‐two Type III inhibitors bind to the allosteric region within the ATP site without influencing ATP binding. Type IV inhibitors interact with the allosteric region external to the kinase domain and do not obstruct ATP binding. These inhibitors function to obstruct the interaction between kinases and substrates. Recently developed Type V kinase inhibitors target both the ATP binding site and the distinct allosteric site. Type VI kinase inhibitors function by establishing covalent bonds with cysteine and other amino acids inside the ATP binding site or other kinase domains. These studies involved in vitro and in silico investigations focused on the development of effective Type V and VI MAPK inhibitors [29, 30, 31, 32].

Given its essential role in regulating proinflammatory signaling networks and the biosynthesis of cytokines such as tumor necrosis factor‐α (TNF‐α) and interleukin‐1β (IL‐1β), p38α MAPK has emerged as a critical therapeutic target for various pathological conditions [33, 34]. Its involvement has been well‐documented in autoimmune and inflammatory diseases, including rheumatoid arthritis [35]. Furthermore, accumulating evidence supports the role of p38α MAPK in neurodegenerative disorders such as Alzheimer's disease [36, 37] and Parkinson's disease [38], as well as in cancer [7, 8], ischemic heart disease [39], and chronic graft‐versus‐host disease [40]. Under stress conditions, p38α MAPK catalyzes the transfer of the γ‐phosphate from ATP to the hydroxyl group of serine and threonine residues in its substrate proteins, thereby initiating key signaling cascades [41].

Dysregulation of MAPK pathways, which regulate cell proliferation, differentiation, and apoptosis, is frequently implicated in cancer progression [42]. Among the three primary MAPK families, ERK (e.g., p44/42) predominantly promotes cell survival and proliferation [43, 44], whereas JNK/SAPK and p38 MAPK are primarily activated by stress signals and are involved in cell‐cycle arrest and apoptosis [45]. Targeting MAPK pathways in combination with conventional anticancer agents has shown promise in enhancing therapeutic efficacy [46, 47].

**Figure 2:**
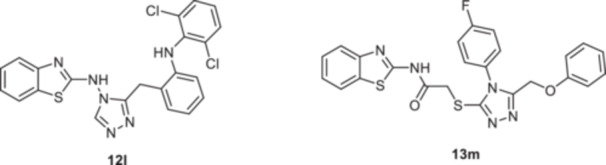
MAPK inhibitors containing a benzothiazole moiety.

Benzothiazole is recognized as a key pharmacophore with a broad spectrum of biological activities, including antimicrobial, anticancer, antioxidant, anti‐inflammatory, anticonvulsant, and antimalarial effects [48]. Among a series of benzothiazole‐containing compounds, compound (**12l**), illustrated in Figure 2, demonstrated the highest potency against p38α MAPK, with an IC_50_ value of 36 nM, surpassing the standard reference SB 203580 (IC_50_: 0.043 ± 0.27 μM). Furthermore, in silico docking studies revealed that compound (**12l**) exhibited stronger interactions with the p38α MAP kinase enzyme compared with SB 203580, suggesting a favorable binding mode [49, 50]. Tariq et al. synthesized a series of novel N‐(benzothiazol‐2‐yl)‐2‐[(5‐(phenoxymethyl)‐4‐aryl‐4*H*‐1,2,4‐triazol‐3‐yl)thio]acetamide derivatives and evaluated their in vitro p38α MAPK inhibition activity using a carrageenan‐induced rat paw edema model. Among them, compound (**13m**) emerged as the most potent inhibitor, with an IC_50_ value of 0.031 ± 0.14 μM, demonstrating superior activity compared to SB 203580 (IC_50_: 0.043 ± 0.14 μM) [51].

This study seeks to create new benzothiazole scaffold‐based compounds that have functional groups known for their anticancer properties, specifically targeting breast cancer growth and inhibiting p38α MAP kinase activity. We assess the synthesized compounds for in vitro antiproliferative efficacy against two cancer cell lines: ER+ breast cancer MCF7 and ER‐ breast cancer MDA‐MB‐231. Additionally, we evaluate the most effective cytotoxic derivatives against the MCF7 cell line for their p38α MAP kinase‐inhibiting properties. Molecular docking experiments are performed to clarify the structural prerequisites for the inhibition of p38α MAP kinase, concentrating on the enzyme's ATP binding site and the allosteric non‐ATP binding site. Consequently, the compounds developed and synthesized in this investigation, exhibiting confirmed suppression of p38α MAP kinase activity, were recognized as possible precursors for Type V MAPK inhibitors.

## 2 Results and Discussion

### 2.1 Chemistry

**Scheme 1:**
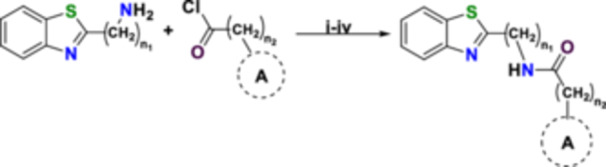
General synthetic route of compounds **1–28**. Reagents and conditions: (i) Dry DCM, Ice path, 2 h; (ii) TEA/DIPEA, Reflux/RT, 24–48 h (iii) Extraction CH_2_Cl_2_ (iv) 1 N HCl, then saturated solution of NaHCO_3_ and finally H_2_O. **n**_**1**_ = 0, 1, **n**_**2**_ = 0, 1, **A** = phenyl, 4‐chlorophenyl, 4‐fluorophenyl, 4‐bromophenyl, 4‐(trifluoromethyl) phenyl, 4‐methoxyphenyl, 3‐methoxyphenyl, 2,5‐dimethoxyphenyl, 4‐methylphenyl, 2‐thiophene, 2‐furan.

The synthesis pathways to benzothiazole derivatives are depicted in Scheme 1. The target compounds (**1–28**) were synthesized via a nucleophilic addition‐elimination reaction, wherein 2‐aminobenzothiazole or 2‐aminomethylbenzothiazole were acylated with benzoyl chloride or phenyl acetyl chloride (or their derivatives) to yield *N*‐(benzo[*d*]thiazol‐2‐yl) substituted benzamides and *N*‐(benzo[*d*]thiazol‐2‐ylmethyl) substituted benzamides, *N*‐(benzo[*d*]thiazol‐2‐yl)‐2‐substituted phenylacetamides and *N*‐(benzo[*d*]thiazol‐2‐ylmethyl)‐2‐substituted phenylacetamides (Table 1).

**Table 1:** Structures of Benzothiazole synthetic compounds caption.

Compounds	n_1_	n_2_	A	Compounds	n_1_	n_2_	A
**1**	0	0	4‐Methoxyphenyl	**15**	1	0	2‐Furan
**2**	0	0	4‐Fluorophenyl	**16**	1	0	4‐Bromophenyl
**3**	0	0	2‐Thiophene	**17**	1	0	4‐Methoxyphenyl
**4**	0	0	2‐Furan	**18**	1	0	4‐Methylphenyl
**5**	0	0	4‐Bromophenyl	**19**	0	1	Phenyl
**6**	0	0	4‐(Trifluoromethyl)phenyl	**20**	0	1	2,5‐dimethoxyphenyl
**7**	0	0	Phenyl	**21**	0	1	3‐Methoxyphenyl
**8**	0	0	4‐Methylphenyl	**22**	0	1	4‐Fluorophenyl
**9**	0	0	4‐Chlorophenyl	**23**	0	1	4‐Chlorophenyl
**10**	1	0	Phenyl	**24**	0	1	4‐methoxyphenyl
**11**	1	0	4‐Chlorophenyl	**25**	0	1	2‐Thiophene
**12**	1	0	4‐Fluorophenyl	**26**	1	1	3‐methoxyphenyl
**13**	1	0	4‐(Trifluoromethyl)phenyl	**27**	1	1	4‐Chlorophenyl
**14**	1	0	2‐Thiophene	**28**	1	1	2‐Thiophene

Spectral data corroborated the structures of the target benzothiazoles (**1–28**). The ^1^H NMR spectra of the amide (–CONH–) proton in compounds **5–9** exhibited a singlet signal at *δ* 12.82–13.18 ppm. The amide (–CONH–) proton was detected in compounds **11–18** at *δ* 9.40–9.79 ppm as a triplet signal. In compounds **19–28**, the two protons of the acetamide spacer (–COCH_2_–) appear as a singlet signal, moved downfield (between δ 3.52–3.97 ppm) due to the deshielding effect of the carbonyl group and the aromatic ring. The remaining two protons of methyl (–CH_2_–) attached to benzothiazole were detected in compounds **26–28** at *δ* 3.97–4.67 ppm as a singlet signal, whereas in compounds **10–18** they appeared at δ 4.85–4.93 ppm as a doublet signal. The ^13^C NMR spectra indicated the existence of a carbonyl group (–CO–) within the δ range of 158.25–175.09 ppm. The methyl carbon spacer (–CH_2_–) is identified by two distinct signals: one in a *δ* range of 37.73–43.99 ppm corresponding to (–COCH_2_–), and the other at 38.37–42.68 ppm associated with (–CH_2_–) linked to benzothiazole.

### 2.2 Biological Activity

#### 2.2.1 In Vitro Antiproliferative Activity and Selectivity

Cell culture experiments were performed to assess the antiproliferative efficacy of the synthesized benzothiazole compounds on the MCF7 and MDA‐MB‐231 cell lines. The anticancer activity of the compounds were compared with the L929 healthy cell line as a control. Doxorubicin and cisplatin, the anticancer agents, were employed as positive controls. The IC_50_ values were determined following 24 h of incubation of the compounds and control drugs in the designated cell lines, as shown in Table 2.

**Table 2:** IC_50_ (µM) values of the synthesized compounds against different cancer cell lines.

Compounds	IC_50_ (µM)			Selectivity	
	MCF7	MDA‐MB‐231	L929	MCF7	MDA‐MB‐231
**1**	11.02 ± 0.27	15.84 ± 0.75	24.54 ± 0.56	2.227	1.549
**2**	11.16 ± 0.49	16.97 ± 0.67	21.02 ± 0.21	1.884	1.239
**3**	15.95 ± 0.46	15.03 ± 0.57	35.24 ± 0.95	2.209	2.345
**4**	11.08 ± 0.08	19.94 ± 0.36	26.03 ± 0.13	2.349	1.305
**5**	12.93 ± 0.37	15.26 ± 0.32	31.02 ± 0.66	2.399	2.033
**6**	13.49 ± 0.37	10.88 ± 0.28	23.90 ± 0.32	1.772	2.197
**7**	12.39 ± 0.97	11.78 ± 0.57	26.90 ± 0.97	2.171	2.284
**8**	4.69 ± 0.05	5.20 ± 0.19	6.44 ± 0.05	1.373	1.238
**9**	3.49 ± 0.32	6.02 ± 0.18	6.43 ± 0.05	1.842	1.068
**10**	5.64 ± 0.34	13.52 ± 0.54	40.82 ± 0.12	7.238	3.019
**11**	15.57 ± 0.65	10.01 ± 0.36	35.87 ± 0.23	2.304	3.583
**12**	21.09 ± 0.46	14.98 ± 0.26	42.98 ± 0.56	2.038	2.869
**13**	12.93 ± 0.86	10.01 ± 0.43	32.76 ± 0.43	2.534	3.273
**14**	10.61 ± 0.16	15.99 ± 0.68	22.24 ± 0.68	2.096	1.391
**15**	8.83 ± 0.43	11.78 ± 0.11	26.76 ± 0.84	3.031	2.272
**16**	14.10 ± 0.95	20.25 ± 0.19	35.76 ± 0.46	2.536	1.766
**17**	11.05 ± 0.23	16.74 ± 0.36	25.09 ± 0.18	2.271	1.499
**18**	11.16 ± 0.75	20.15 ± 0.73	29.06 ± 0.52	2.604	1.442
**19**	6.19 ± 1.05	10.74 ± 0.03	93.91 ± 1.90	15.171	8.744
**20**	14.69 ± 0.15	16.07 ± 0.12	87.80 ± 1.29	5.977	5.464
**21**	7.09 ± 0.02	6.42 ± 0.07	66.32 ± 1.82	9.354	10.330
**22**	5.05 ± 0.01	3.80 ± 0.28	210.57 ± 1.67	41.697	55.413
**23**	8.24 ± 0.19	7.04 ± 0.18	53.97 ± 1.08	6.550	7.666
**24**	12.65 ± 0.18	12.92 ± 0.19	81.47 ± 1.07	6.440	6.306
**25**	13.21 ± 0.03	14.38 ± 0.18	134.60 ± 1.83	10.189	9.360
**26**	13.52 ± 0.35	15.78 ± 0.52	27.67 ± 0.78	2.047	1.753
**27**	12.34 ± 0.96	14.67 ± 0.63	32.90 ± 0.35	2.666	2.243
**28**	11.11 ± 0.37	18.76 ± 0.23	31.04 ± 0.33	2.794	1.655
Doxorubicin	5.80 ± 0.15	9.27 ± 0.21	23.96 ± 1.07	4.131	2.584
Cisplatin	7.03 ± 0.04	30.11 ± 1.39	35.98 ± 2.01	5.118	1.195

Table 2 indicates that compound **9** had the highest potency against the MCF7 breast cancer cell line, with an IC_50_ value of 3.49 ± 0.32 µM. Compounds **8**, **10**, and **22** exhibited notable action relative to the standard drugs (doxorubicin and cisplatin), with IC_50_ values of 4.69 ± 0.05, 5.64 ± 0.34, and 5.05 ± 0.01 µM, respectively. Compounds **15**, **19**, **21**, and **23** demonstrated IC_50_ values similar to those of the standard drugs against the MCF7 cancer cell line.

Compound **22** exhibited the highest efficacy against the MDA‐MB‐231 breast cancer cell line, demonstrating an IC_50_ value of 3.80 ± 0.28 µM. Compounds **8**, **9**, **21**, and **23** exhibited lower IC_50_ values than the reference drugs (doxorubicin and cisplatin) against the MDA‐MB‐231 breast cancer cell line, with IC_50_ values of 5.20 ± 0.19, 6.02 ± 0.18, 6.42 ± 0.07, and 7.04 ± 0.18 µM, respectively.

Selectivity denotes the extent to which a chemical specifically inhibits a biological target while exerting low or no influence on other targets. The selectivity index of the compounds was ascertained based on the findings of the MTT study (Table 2). The selectivity index values were determined using the IC_50_ values (24‐h) of the MCF7 and MDA‐MB‐231 cell lines in relation to L‐929 cells, employing the formula: Selectivity index (S.I) = Healthy cell IC_50_ value/Cancerous cell IC_50_ value.

Upon evaluating the IC_50_ values of all compounds using this methodology, compound **22** exhibited the best selectivity index, with values of 41.697 against the MCF7 cell line and 55.413 against the MDA‐MB‐231 cell line. Compounds **19–25**, including phenylacetamide sidechains, exhibited elevated selectivity index values (5.464–55.413), signifying exceptional specificity.

#### 2.2.2 Evaluation of p38 MAPKα Activity of the Selected Most Active Compounds

The levels of p38 MAPKα in the 16 most potent compounds against the MCF7 cell line were assessed. To account for variations in protein quantities among the cells. The total protein concentrations in cells subjected to the drugs were quantified. The p38 MAPKα levels were adjusted by dividing by the protein levels, yielding results represented as ng/μg protein (Table 3).

**Table 3:** Compounds total p38α MAPK level.

Compound	Total p38α MAPK levels (ng/μg protein)	Compound	Total p38α MAPK levels (ng/μg protein)
**1**	7.367 ± 1.291	**15**	0.155 ± 0.045
**2**	3.879 ± 0.868	**17**	6.411 ± 1.604
**3**	0.430 ± 0.005	**18**	1.340 ± 0.154
**4**	8.529 ± 0.9	**19**	0.112 ± 0.001
**8**	9.541 ± 0.099	**21**	1.333 ± 0.007
**9**	0.166 ± 0.015	**22**	3.952 ± 0.311
**10**	0.535 ± 0.079	**23**	2.331 ± 1.241
**14**	0.199 ± 0.011	**28**	2.542 ± 0.228

The overall p38 MAPKα concentration for compound **19**, an effective drug against the MCF7 cell line, was 0.112 ng/μg protein, exhibiting a selectivity index of 15.171 against MCF7. This demonstrates the antiproliferative impact of compound **19** on MCF7 cells. Moreover, literature indicates that p38 MAPK levels are generally elevated in MCF7 cells relative to healthy cells. The reduced levels of p38α MAPK in highly selective drugs strongly indicate that their antiproliferative activities may be facilitated by p38 MAPK inhibition. Compounds **9**, **14**, **15**, and **19**, which revealed substantial antiproliferative actions, displayed markedly low levels of p38 MAPK.

### 2.3 Molecular Docking

In silico investigations sought to ascertain the binding conformations of novel benzothiazole derivatives exhibiting p38α MAPK inhibitory activities and to classify the kind of inhibitor. The affinity of possible derivatives for both the ATP binding pocket and the recently identified lipid binding site (closer to the MAPK insert site) was examined.

**Figure 3:**
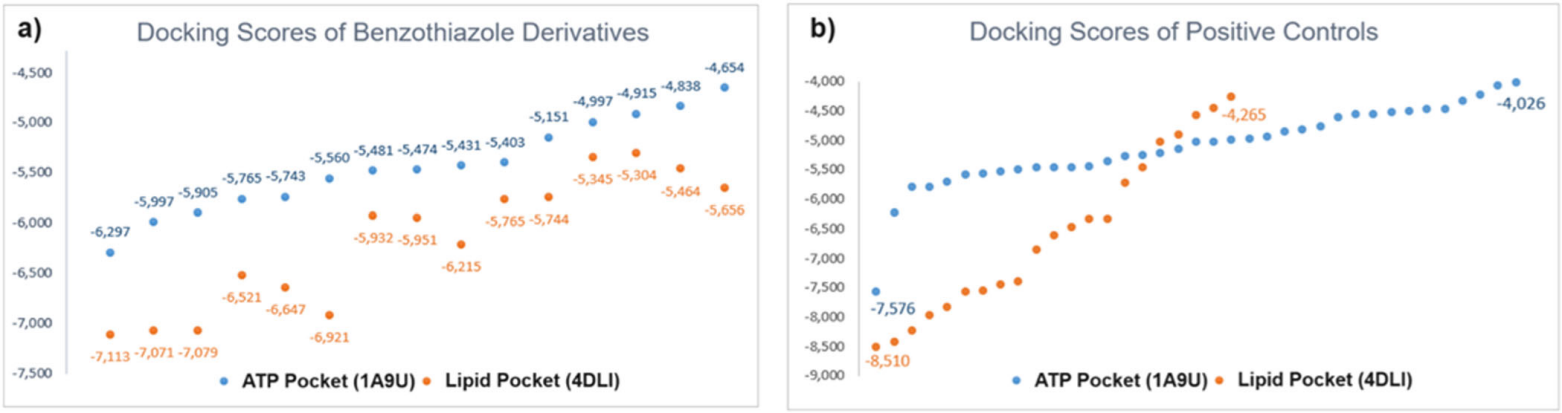
Docking graphs of benzothiazole derivatives (a) and positive controls (b) in the ATP pocket and Lipid Pocket in the MAPK p38 crystal structures.

Molecular docking studies were conducted to ascertain the affinities and binding conformations of the most active benzothiazole derivatives to the ATP pocket and lipid pocket of p38 MAPK. The docking scores of the compounds in the ATP binding region varied from –4.654 to –6.297 kcal/mol, while those in the lipid‐binding region ranged from –5.304 to –7.113 kcal/mol (Figure 3a, Table 4).

**Table 4:** Docking scores and MM‐GBSA binding affinity of benzothiazole derivatives (kcal/mol) against target proteins (1A9U and 4DLI).

	1A9U		4DLI			1A9U		4DLI	
Compound	Docking score	MM‐GBSA binding affinity	Docking score	MM‐GBSA binding affinity	Compound	Docking score	MM‐GBSA binding affinity	Docking score	MM‐GBSA binding affinity
**1**	−4.838	−29.84	−5.464	−18.32	**15**	−5.905	−41.92	−7.079	−38.02
**2**	−5.151	−33.99	−5.744	−28.64	**17**	−4.997	−31.47	−5.345	−21.83
**3**	−5.431	−39.21	−6.215	−32.14	**18**	−5.403	−36.24	−5.765	−30.65
**4**	−4.654	−26.77	−5.656	−25.82	**19**	−6.297	−44.17	−7.113	−42.55
**8**	−4.915	−26.50	−5.304	−23.64	**21**	−5.481	−30.84	−5.932	−31.11
**9**	−5.743	−36.28	−6.647	−38.65	**22**	−5.474	−33.55	−5.951	−27.17
**10**	−5.765	−34.06	−6.521	−33.69	**23**	−5.650	−36.58	−6.384	−32.40
**14**	−5.997	−49.16	−7.071	−37.37	**28**	−5.560	−34.48	−6.921	−38.50

To validate the molecular docking analysis, 37 established ligands (positive controls) for the ATP pocket (1A9U) and 21 established ligands (positive controls) for the lipid binding site (4DLI) were recognized. The identified ligands were obtained from the PDB files and docked to the target proteins before modeling investigations (Supporting Information S2: Table S1, S2). The binding energies were computed. The docking scores of the ligands for 1A9U and 4DLI ranged from –4.026 to –7.576 kcal/mol and –4.265 to –8.510 kcal/mol, respectively, serving as reference values (Figure 3b). For the validation of molecular docking studies, native ligands found in 4DLI and 1A9U structures were removed, minimized, and redocked from the IRG and SB2 structures, respectively. The RMSD values of IRG and SB2 were found to be 1.52 Å and 0.98 Å, respectively.

Upon comparison of the docking scores of the compounds with those of the reference compounds, it was noted that the compounds demonstrated a strong affinity for both the ATP‐binding site and the lipid‐binding site. Moreover, all compounds exhibited a greater affinity for the lipid binding region compared with the ATP binding region. This indicates that these compounds may serve as Type V MAPK inhibitors due to their robust affinity for both the ATP and lipophilic regions.

**Figure 4:**
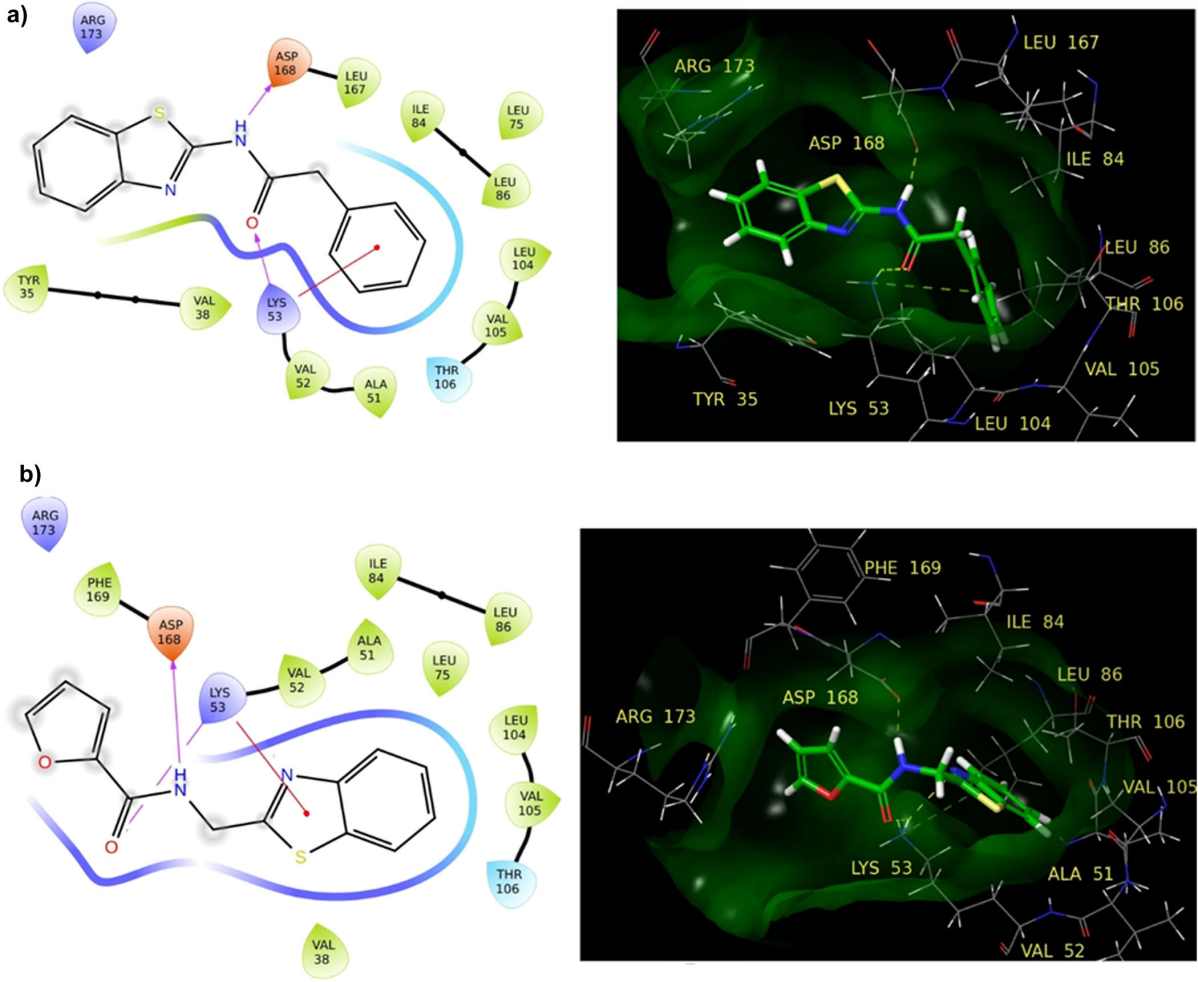
2D and 3D interactions in the ATP binding domain of **19** (a) and **15** (b) (1A9U PDB encoded protein).

Among the benzothiazole derivatives, compound **19** exhibited the greatest affinity for both targets. Essential components in the phosphate binding mechanism comprise the DFG motif (Asp‐Phe‐Gly) and E71 within the ATP binding site (1A9U). The DFG‐in state signifies the capacity to compete with ATP. Compounds **19** and **15** exhibited significant activity by forming hydrogen bonds with Asp168 and Lys53. The bonding were facilitated by the amide groups in their structures (Figure 4). Furthermore, both compounds demonstrated a pi–cation interaction with Lys53 in the active site.

**Figure 5:**
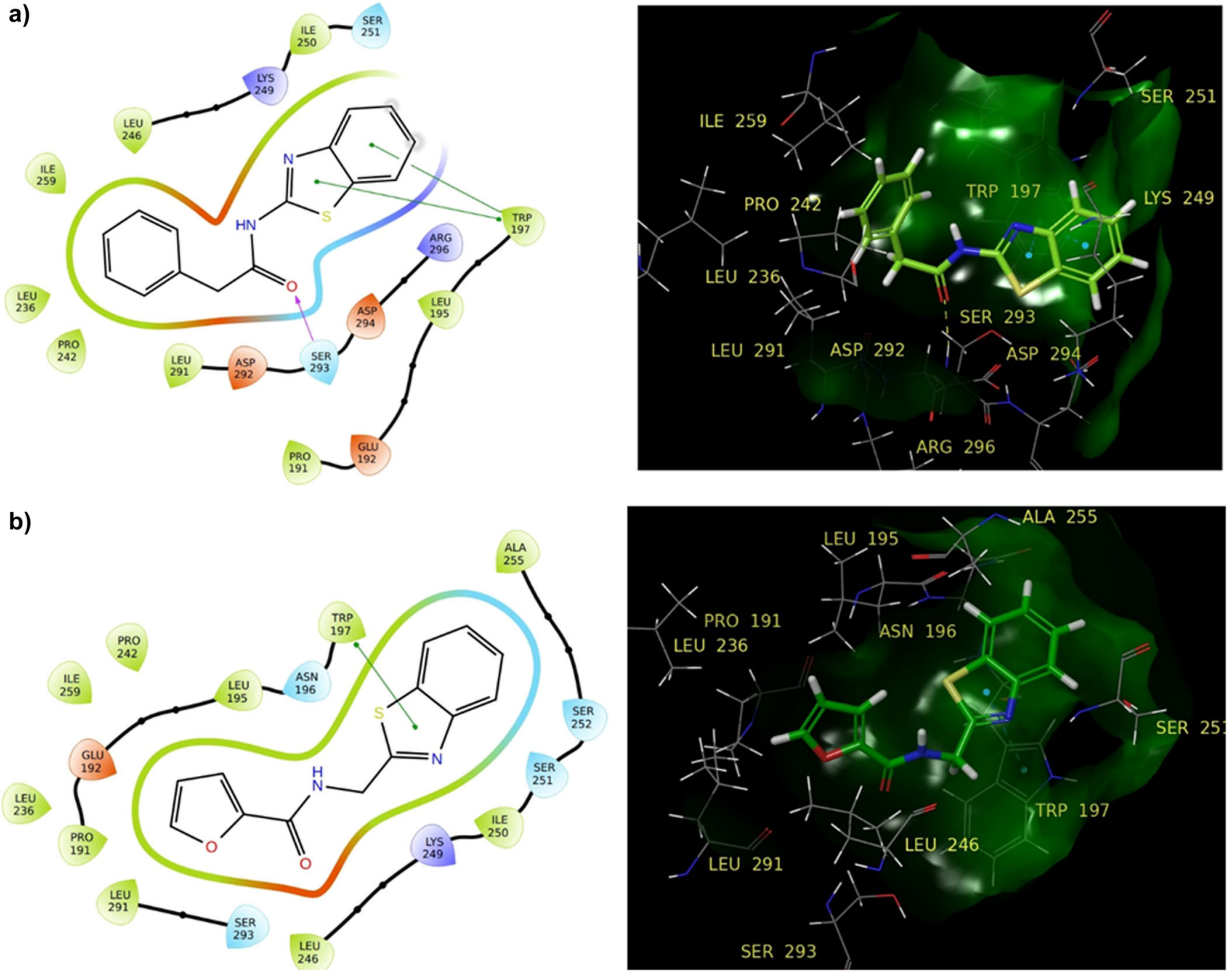
2D and 3D interactions in the lipid domain of **19** (a) and **15** (b) (4DLI PDB encoded protein).

In the DFG‐out conformation, when the receptor is unresponsive to substrate binding, chemicals associate with the hydrophobic pocket. This pocket comprises hydrophobic residues, including many leucines. Trp197 is one of the key residues in the lipid pocket. Compounds **19** and **15** established a pi–pi stacking interaction with Trp197 via the benzothiazole ring. Compound **19**, including a phenyl ring and a benzothiazole ring, engages in hydrophobic interactions with Pro191, Leu195, Leu236, Pro242, Leu246, Ile250, Ile259, and Leu291 within the lipophilic pocket. Compound **15** similarly interacts with the pocket by establishing lipophilic contacts with Pro191, Leu195, Leu236, Pro242, Leu246, Ile250, Ala255, Ile259, and Leu291 in the lipid area via its furan and benzothiazole rings (Figure 5).

**Figure 6:**
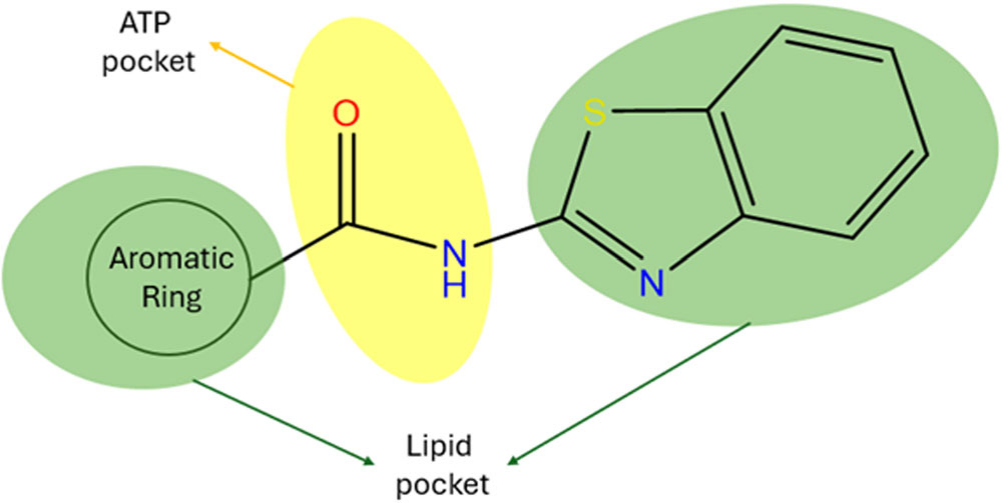
Effective groups in the ATP and lipid binding site of benzothiazole derivatives. Green: lipid pocket, yellow: ATP pocket.

The amide group in the structures of benzothiazole derivatives effectively interacts with the ATP site, while the benzothiazole ring and aromatic substituents engage with the lipid pocket (Figure 6). The benzothiazole ring, aromatic rings, and amide group are essential for the development of a p38α MAPK inhibitor.

### 2.4 MM/GBSA Calculations

The MM‐GBSA analysis was used to find out how the protein and ligand complexes bind to each other freely. MM/GBSA scores of ligands were calculated separately for 4DLI and 1A9U. It was found that ligands' binding energies in 4DLI complexes were between –42.55 kcal/mol and –21.83 kcal/mol, while they were between –49.17 kcal/mol and –26.50 kcal/mol in 1A9U complexes. It can be said that docking scores and binding affinities for ligands are generally in agreement. It was determined that compounds **19**, **14**, and **15** had high binding affinities towards 1A9U, while **19**, **15**, **14**, **28**, and **29** had high affinities toward 4DLI.

### 2.5 Molecular Dynamics Simulation

**Figure 7:**
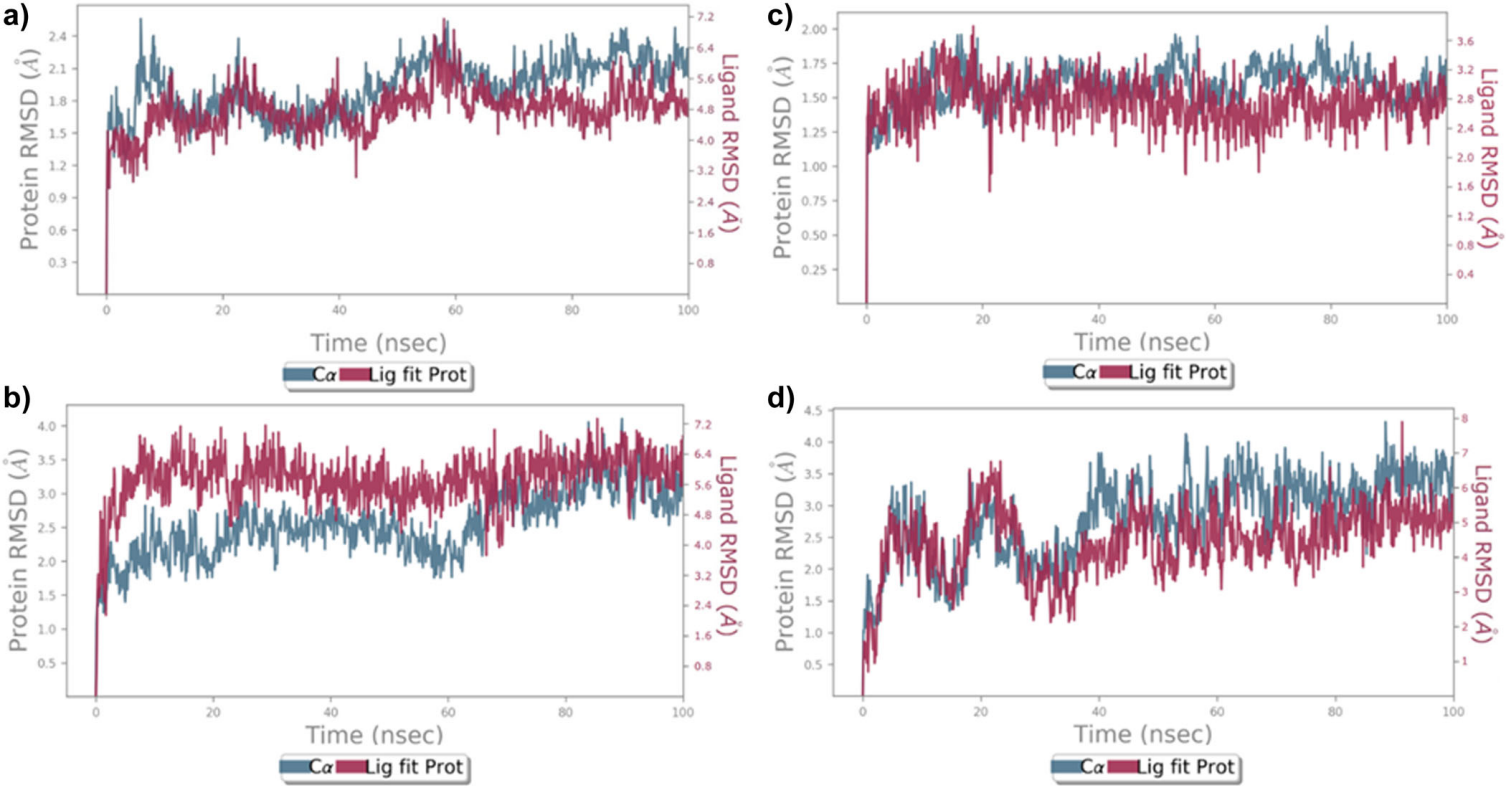
RMSD plots of complex p38α MAPK with **19** and **15** for 100‐ns MD simulations. (a) **19** in inside p38α ATP pocket (1A9U). (b) **19** in inside p38α lipit pocket (4DLI). (c) **15** in inside p38α ATP pocket (1A9U). (d) **15** in inside p38α lipit pocket (4DLI). Blue: Cα (RMSD evolution of the protein). Pink: Lig fit Prot (RMSD of the ligand when the protein–ligand complex).

To verify the molecular modeling studies and ascertain whether the inhibitory compounds interact with the ATP binding site and lipid area of p38α MAP kinase, 100 ns molecular dynamics simulations were conducted (Figure 7). The average RMSD values for compound **19** in the p38α ATP pocket (1A9U) and lipid pocket (4DLI) are 3.8 Å and 3.3 Å, respectively. Compound **19** exhibited stability for 100 ns in both 1A9U and 4DLI. The RMSD value of compound **19** in 1A9U stabilized after the 60th ns, exhibiting an average RMSD of 2 Å from the 60th ns to the conclusion of the simulation. Compound **19** exhibited stability in 4DLI until the conclusion of the 100th ns, undergoing a conformational shift after the 5th ns, resulting in an average RMSD value of 3 Å.

The average RMSD values of compound **15** in the p38α ATP pocket (1A9U) and lipid pocket (4DLI) areas are 2 Å and 5 Å, respectively. Compound **15** demonstrated stability in 1A9U throughout the simulation. Compound **15** entered the hydrophobic pocket of 4DLI due to a conformational alteration during the 48th nanosecond and maintained stability in this location until the simulation concluded. Subsequent to 48 ns, the RMSD plot approximately attained a plateau value. The average RMSD value was determined to be 3 Å between 48 and 100 ns.

Radius of Gyration (rGyr) is an important MD simulation parameter that evaluates the structural compactness and stability of a molecule (protein, ligand complex, etc.) over time. In this study, the effect of the complexes formed by the compounds with 4DLI and 1A9U proteins on rGyr was investigated (Supporting Information S2: Figure S1). The rGyr values of compound **19**, in the 1A9U (a) and 4DLI (b) complexes were observed in the range of 3.6–4.0 Å and 3.45–3.75 Å, respectively, throughout the simulation. Similarly, the rGyr values of compound **15** in 1A9U (c) and 4DLI (d) varied in the range of 3.60–3.90 Å and 3.45–3.37 Å, respectively, during the 100 ns simulation period. When the Rg graphs are evaluated, it is seen that the compounds exhibit a more stable structure in the 1A9U complex compared with 4DLI. In general, it was determined that the rGyr fluctuations of the compounds over time were within acceptable limits and their structures remained stable throughout the simulation.

Solvent Accessible Surface Area (SASA) analysis is an important parameter that provides information about the stability of ligand–protein complexes by determining the surface area of the molecules interacting with the solvent (water). In this study, the SASA values of compounds **19** and **15** in 4DLI and 1A9U proteins were analyzed (Supporting Information S2: Figure S2). The SASA values of compound **19** in the 1A9U (a) and 4DLI (b) complexes varied between approximately 10 and 60 Å^2^ during the simulation. These fluctuations indicate that the compound is stable in two different regions within the protein. In the SASA analysis of compound **15**, the SASA values in the 1A9U (c) and 4DLI (d) complexes varied between 10 and 40 Å^2^ and 20–90 Å^2^, respectively. While it was observed that the complex formed with the 1A9U protein remained stable for 100 ns, it was determined that the fluctuations decreased and the ligand became stable after the 50th nanosecond of the simulation for the complex formed with 4DLI.

## 3 Conclusions

Twenty‐eight compounds based on a benzothiazole scaffold were developed, synthesized, and assessed for their antiproliferative efficacy against breast cancer. Their antiproliferative properties were assessed via the MTT test and compared with the standard drugs doxorubicin and cisplatin. Thereafter, the compounds with the greatest activity against the MCF7 cell line were chosen, and their inhibitory effects on the p38α MAPK enzyme were examined. Compounds **15** and **19** exhibited similar IC_50_ values (8.83 ± 0.43 and 6.19 ± 1.05 µM, respectively) when compared with the standard drugs against the MCF7 cancer cell line. Furthermore, compound **19** demonstrated elevated selectivity index values of 15.171 for the MCF7 cell line and 8.744 for the MDA‐MB‐231 cell line, signifying exceptional specificity relative to the standard drugs. The total p38 MAPKα levels for compounds **19** and **15** were the lowest, measuring 0.112 and 0.155 ng/μg protein, respectively.

The molecular docking studies of compounds **19** and **15** demonstrated their significant binding affinities for p38α MAPK. The evaluated compounds for docking exhibited greater affinities for the lipid binding region compared with the ATP binding region. Consequently, these compounds may function as Type V MAPK inhibitors owing to their robust affinity for both the ATP site and the lipophilic domain. The docking results indicated that the amide group in the benzothiazole derivatives successfully interacts with the ATP site, while the benzothiazole ring and aromatic substituents effectively engage with the lipid pocket.

Molecular dynamics simulations conducted over 100 ns demonstrated that compounds **19** and **15** exhibit stability within both the ATP binding domain and the lipid domain of p38α MAPK. Molecular docking and molecular dynamics simulation results indicate that the elevated levels of p38 MAPKα in MCF7 cells, compared with healthy cells, suggest that the low p38 MAPKα levels of compounds **19** and **15** provide compelling evidence for their antiproliferative effects via p38α MAPK inhibition.

Among the tested compounds, **7**, **9**, **14**, **15**, **19**, **21**, **22**, and **25** exhibited significant antiproliferative activity, with compound **22** demonstrating particularly notable potency (Table 2). However, variations in p38α MAPK inhibition levels were observed among these compounds. Only compounds **9**, **14**, **15**, and **19** displayed high inhibitory activity against p38α MAPK (Table 3).

A structure–activity relationship (SAR) analysis revealed that compounds with a substituted aromatic ring in their side chains exhibited strong antiproliferative activity, whereas high p38α MAPK inhibition levels were linked to nonsubstituted compounds. Molecular docking studies further support this finding. Additionally, except for compound **9**, the most active compounds contained a single CH_2_ bridge, suggesting that this structural feature contributes to enhanced flexibility and, consequently, better p38α MAPK inhibition. Notably, when this bridge was positioned on the aromatic ring rather than the benzothiazole core, it appeared to be more compatible with the p38α MAPK binding sites. However, in compounds **26–28**, which contained CH_2_ bridges on both sides of the amide group, no significant activity was observed, indicating a potential limit to the flexibility required for effective binding.

The benzothiazole core, amide group, and aromatic ring were identified as key pharmacophoric elements for p38α MAPK inhibition, effectively interacting with both ATP and lipid binding sites. While non‐substituted aromatic rings still retained activity, the phenyl ring showed superior binding compatibility.

The strong activity and high selectivity of compound **22** against both breast cancer cell lines is notable. However, p38α MAPK does not appear to be a suitable target for this compound, emphasizing the significant role of the aromatic substituent in p38α MAPK activity. Further investigations are needed to explore alternative molecular targets and evaluate compound **22** as a potential lead compound.

In conclusion, modifications to the benzothiazole scaffold present a promising strategy for the development of novel p38α MAPK inhibitors targeting breast cancer cells.

## 4 Experimental

### 4.1 Chemistry

#### 4.1.1 General

All reagents used were commercially available unless otherwise specified, and all solvents were distilled before use. Analytical thin layer chromatography (TLC) was performed on Merck silica gel 60 F254. Fluka Silica gel 60 (0.063–0.2 mm) was used for column chromatography. The visualization of TLC was accomplished with UV light (254 nm) and by staining with ethanolic PMA (phosphomolybdic acid) solution. Melting points were measured with Gallenkamp melting point devices. IR spectra were recorded on Perkin–Elmer Spectrum One FT‐IR Spectrometer. ^1^H NMR and ^13^C NMR spectra (see the Supporting Information) were recorded on 400 (100)‐MHz Varian and Bruker Spectrometer and are reported in δ units with SiMe_4_ as the internal standard. Mass spectra were recorded on an Agilent 6530 Accurate‐Mass Q‐TOF‐LC/MS.

The InChI codes of the investigated compounds, together with some biological activity data, are provided as Supporting Information.

#### 4.1.2 General Procedure for the Synthesis of 2‐Aminomethylbenzothiazole

2‐Aminothiophenol (2 mmol) and glycine (2.2 mmol, 0.165 g) were combined with an adequate amount of polyphosphoric acid (8 g) to achieve a homogenous mixture. The mixture was heated to 180°C, and the resultant solution was stirred at the same, temperature. The reaction's development was monitored using TLC. Upon cooling the reaction mixture to ambient temperature, it was subsequently transferred to an ice bath, neutralized with the addition of NaHCO_3_ (pH = 8). Extraction was conducted utilizing dichloromethane (3 × 15 mL). The organic phase was separated, treated with MgSO_4_, filtered, and the solvent was removed at reduced pressure. The solid product was utilized immediately to synthesize amide without additional purification.

#### 4.1.3 General Synthesis Method for Compounds **1–9**, **19–25**

2‐Aminobenzothiazole (1.1 mmol) was dissolved in dichloromethane (15 mL). Benzoyl chloride or its derivatives (2‐furoyl chloride, 2‐thiophenecarbonyl chloride, phenylacetyl chloride, or derivatives such as 2‐thiopheneacetyl chloride) (1 mmol) were added dropwise to the solution during a duration of 10 min at 0°C. The reaction mixture was stirred at a constant temperature for 2 h. Subsequently, five drops of triethylamine (TEA) or diisopropylethylamine (DIPEA) were added at ambient temperature. The reaction mixture was subjected to reflux for 24 h. The reaction's completion was monitored using TLC. Upon completion, the reaction stopped and 25 mL of dichloromethane was added. The organic phase underwent washing with 1 N HCl (3 × 20 mL), succeeded by a saturated NaHCO_3_ solution (2 × 15 mL) and 15 mL of distilled water. The organic phase was separated, desiccated using MgSO_4_, filtered, and the solvent was removed under reduced pressure. The solid product was further purified using column chromatography with a suitable mobile phase (n‐hexane/ethyl acetate). The final products underwent recrystallization with a suitable solvent.

*N*‐(Benzo[*d*]thiazol‐2‐yl)‐4‐methoxybenzamide (**1**): White crystals, m.p.: 237.2°C, Yield: 78.6%. Rf = 0.6 Chloroform/Methanol (9.5:0.5) ^1^H NMR (400 MHz, CDCl_3_) *δ* = 7.91–7.88 (m, 1H, Ar‐H), 7.88–7.87 (m, 1H, Ar‐H), 7.80–7.74 (m, 1H, Ar‐H), 7.31–7.24 (m, 1H, Ar‐H), 7.23–7.18 (m, 2H, Ar‐H), 6.82–6.80 (m, 1H, Ar‐H), 6.79–6.77 (m, 1H, Ar‐H), 3.74 ppm (s, 3H, –OCH_3_). ^13^C NMR (100 MHz, CDCl_3_) *δ* = 165.15, 163.55, 159.77, 147.93, 132.04, 129.94, 126.07, 124.14, 123.89, 121.37, 120.71, 114.30, 55.53 ppm. IR (cm^−1^): 3450, 3015, 2969, 2944, 1739, 1537, 1515, 1439, 1369, 1228, 1216. HRMS: *m/z* [M+H]^+^ calcd. For C_15_H_12_N_2_O_2_S: 285.0698, Found: 285.0683.

*N*‐(Benzo[*d*]thiazol‐2‐yl)‐4‐fluorobenzamide (**2**): White crystals, m.p.: 212°C, Yield: 82.2%. Rf = 0.58 Chloroform/Methanol (9.5:0.5) ^1^H NMR (400 MHz, CDCl_3_) *δ* = 7.94 (dd, *J* = 9.0, 5.2 Hz, 2H, Ar‐H), 7.78 (dd, J = 5.2, 2.7 Hz, 1H, Ar‐H), 7.24 (dd, *J* = 9.0, 5.2 Hz, 3H, Ar‐H), 7.00 ppm (t, *J* = 9.0 Hz, 2H, Ar‐H). ^13^C NMR (100 MHz, CDCl_3_) *δ* = 166.94, 164.90, 164.41, 159.86, 147.60, 131.90, 130.57, 128.28, 126.22, 124.19, 121.50, 120.55, 116.33, 116.11 ppm. IR (cm^−1^): 3450, 3015, 2969, 2944, 1739, 1598, 1544, 1502, 1455, 1444, 1369, 1216, 753. HRMS: *m/z* [M+H]^+^ calcd. For C_14_H_9_FN_2_OS: 273.0498, Found: 273.0483.

*N*‐(Benzo[*d*]thiazol‐2‐yl) thiophene‐2‐carboxamide (**3**): Yellow crystals, m.p.: 232°C, Yield: 94%. Rf = 0.7 Chloroform/Methanol (9.5:0.5) ^1^H NMR (400 MHz, CDCl_3_) *δ* = 7.80–7.73 (m, 1H, Ar‐H), 7.66 (dd, *J* = 3.8, 1.0 Hz, 1H, Ar‐H), 7.54 (dd, J = 4.9, 1.0 Hz, 1H, Ar‐H), 7.37–7.30 (m, 1H, Ar‐H), 7.27–7.19 (m, 2H, Ar‐H), 6.92 ppm (dd, *J* = 4.9, 3.8 Hz, 1H, Ar‐H). ^13^C NMR (100 MHz, CDCl_3_) *δ* = 160.51, 159.86, 147.49, 136.60, 133.01, 132.00, 130.85, 128.25, 126.26, 124.09, 121.50, 120.56 ppm. IR (cm^−1^): 2969, 1738, 1659, 1598, 1548, 1454, 1440, 1406, 1292, 1276, 852, 715. HRMS: *m/z* [M+H]^+^ calcd. For C_12_H_8_N_2_OS_2_: 261.0156, Found: 261.0145.

*N*‐(Benzo[*d*]thiazol‐2‐yl) furan‐2‐carboxamide (**4**): Light Yellow crystals, m.p.: 190°C, Yield: 97%. Rf = 0.61 Chloroform/Methanol (9.5:0.5).^1^H NMR (400 MHz, CDCl_3_) *δ* = 7.78 (dd, *J* = 7.9, 0.6 Hz, 1H, Ar‐H), 7.67–7.61 (m, 1H, Ar‐H), 7.36–7.31 (m, 1H, Ar‐H), 7.29–7.22 (m, 2H, Ar‐H), 7.18 (d, *J* = 1.5 Hz, 1H, Ar‐H), 6.45 ppm (dd, *J* = 3.5, 1.5 Hz, 1H, Ar‐H). ^13^C NMR (100 MHz, CDCl_3_) *δ* = 158.25, 155.87, 148.35, 145.94, 145.67, 132.29, 126.19, 124.04, 121.39, 121.10, 117.64, 112.89 ppm. IR (cm^−1^): 3450, 3015, 2969, 2946, 1739, 1661, 1533, 1439, 1365, 1285, 1216. HRMS: *m/z* [M+H]^+^ calcd. For C_12_H_8_N_2_O_2_S: 245.0385, Found: 245.0369.

*N*‐(Benzo[*d*]thiazol‐2‐yl)‐4‐bromobenzamide (**5**): White crystals, m.p.: 225°C, Yield: 65%. Rf = 0.54 Chloroform/Methanol (9.5:0.5).^1^H NMR (400 MHz, DMSO‐d_6_) *δ* = 13.02 (s, 1H, –NHCO–), 8.14–8.06 (m, 2H, Ar‐H), 8.04 (d, *J* = 7.8 Hz, 1H, Ar‐H), 7.86–7.76 (m, 3H, Ar‐H), 7.54–7.46 (m, 1H, Ar‐H), 7.41–7.33 ppm (m, 1H, Ar‐H). ^13^C NMR (100 MHz, DMSO‐d_6_) *δ* = 165.40, 165.38, 158.97, 131.67, 131.37, 131.27, 130.39, 126.81, 126.23, 123.75, 121.78, 120.25, 120.19 ppm. IR (cm^−1^): 3450, 3015, 2969, 1739, 1676, 1544, 1439, 1365, 1228, 1216, 1063. HRMS: *m/z* [M+H]^+^ calcd. For C_14_H_9_BrN_2_OS: 332.9697, Found: 332.9679.

*N*‐(Benzo[*d*]thiazol‐2‐yl)‐4‐(trifluoromethyl) benzamide (**6**): White crystals, m.p.: 221°C, Yield: 97.6%. Rf = 0.75 Chloroform/Methanol (9.5:0.5).^1^H NMR (400 MHz, DMSO‐d_6_) *δ* = 13.18 (s, 1H, –NHCO–), 8.34 (d, *J* = 8.0 Hz, 2H, Ar‐H), 8.04 (d, J = 8.0 Hz, 1H, Ar‐H), 7.95 (d, *J* = 8.0 Hz, 2H, Ar‐H), 7.81 (d, *J* = 8.0 Hz, 1H, Ar‐H), 7.54–7.46 (m, 1H, Ar‐H), 7.42–7.33 ppm (m, 1H, Ar‐H). ^13^C NMR (100 MHz, DMSO‐d_6_) *δ* = 165.26, 136.08, 132.45, 132.13, 131.24, 129.27, 127.85, 126.30, 125.50, 125.14, 123.84, 122.43, 121.84, 119.72, 48.58 ppm. IR (cm^−1^): 3430, 3015, 2969, 2947, 1739, 1674, 1548, 1455, 1407, 1365, 1228, 1216, 768. HRMS: *m/z* [M+H]^+^ calcd. For C_15_H_9_F_3_N_2_OS: 323.0466, Found: 323.0451.

*N*‐(Benzo[*d*]thiazol‐2‐yl) benzamide (**7**): Brown crystals, m.p.: 199°C, Yield: 80% Rf = 0.48 Chloroform/Methanol (9.5:0.5).^1^H NMR (400 MHz, DMSO‐d_6_) *δ* = 12.91 (s, 1H, –NHCO–), 8.18 (t, *J* = 1.6 Hz, 1H, Ar‐H), 8.16 (t, *J* = 1.6 Hz, 1H, Ar‐H), 8.05 (d, *J* = 7.8 Hz, 1H, Ar‐H), 7.81 (d, *J* = 7.8 Hz, 1H, Ar‐H), 7.72–7.66 (m, 1H, Ar‐H), 7.62–7.57 (m, 2H, Ar‐H), 7.50 (ddd, *J* = 8.2, 7.3, 1.2 Hz, 1H, Ar‐H), 7.37 ppm (ddd, *J* = 8.2, 7.3, 1.2 Hz, 1H, Ar‐H). ^13^C NMR (100 MHz, DMSO‐d_6_) *δ* = 166.09, 166.06, 158.93, 148.21, 132.84, 131.98, 131.47, 128.62, 128.33, 126.17, 123.67, 121.71, 120.30, 120.27 ppm. IR (cm^−1^): 3163, 3055, 2946, 2602, 1671, 1596, 1549, 1475, 1463, 1439, 1315, 1274, 1116. HRMS: *m/z* [M+H]^+^ calcd. For C_14_H_10_N_2_OS: 255.0592, Found: 255.0580.

*N*‐(Benzo[*d*]thiazol‐2‐yl)‐4‐methylbenzamide (**8**): White crystals, m.p.: 185°C, Yield: 70% Rf = 0.51 Chloroform/Methanol (9.5:0.5).^1^H NMR (400 MHz, DMSO‐d_6_) *δ* = 12.82 (s, 1H, –NHCO–), 8.09 (s, 1H, Ar‐H), 8.07 (s, 1H, Ar‐H), 8.04 (d, *J* = 7.9 Hz, 1H, Ar‐H), 7.81 (d, J = 7.9 Hz, 1H, Ar‐H), 7.49 (ddd, *J* = 7.9, 7.3, 1.3 Hz, 1H, Ar‐H), 7.40 (d, J = 7.9 Hz, 2H, Ar‐H), 7.38–7.34 (m, 1H, Ar‐H), 2.42 ppm (s, 3H‐CH_3_). ^13^C NMR (100 MHz, DMSO‐d_6_) *δ* = 165.85, 158.93, 148.43, 143.17, 131.52, 129.18, 128.35, 126.12, 123.60, 121.66, 120.26, 21.08 ppm. IR (cm^−1^): 3200, 2916, 1667, 1595, 1532, 1515, 1442, 1273, 1183, 1130, 1099, 761, 749. HRMS: *m/z* [M+H]^+^ calcd. For C_15_H_12_N_2_OS: 269.0749, Found: 269.0732.

*N*‐(Benzo[*d*]thiazol‐2‐yl)‐4‐chlorobenzamide (**9**): White crystals, m.p.: 212°C, Yield: 47% Rf = 0.71 Chloroform/Methanol (9.5:0.5) ^1^H NMR (400 MHz, DMSO‐d_6_) *δ* = 12.99 (s, 1H,–NHCO–), 8.20–8.18 (m, 1H, Ar‐H), 8.17–8.16 (m, 1H, Ar‐H), 8.05 (d, *J* = 7.3 Hz, 1H, Ar‐H), 7.81 (d, J = 8.2 Hz, 1H, Ar‐H), 7.69–7.67 (m, 1H, Ar‐H), 7.67–7.65 (m, 1H, Ar‐H), 7.50 (ddd, *J* = 8.2, 7.3, 1.2 Hz, 1H, Ar‐H), 7.37 ppm (ddd, *J* = 8.2, 7.3, 1.2 Hz, 1H, Ar‐H). ^13^C NMR (100 MHz, DMSO‐d_6_) δ = 165.33, 159.14, 147.87, 137.74, 131.35, 130.95, 130.26, 128.71, 128.32, 128.29, 126.22, 123.73, 121.76, 120.13 ppm. IR (cm^−1^): 2970, 1675, 1589, 1575, 1547, 1487, 1453, 1438, 1252, 867, 832, 730. HRMS: *m/z* [M+H]^+^ calcd. For C_14_H_9_ClN_2_OS: 289.0202, Found: 289.0195.

*N*‐(Benzo[*d*]thiazol‐2‐yl)‐2‐phenylacetamide (**19**): Yellow crystals. Yield:73.28%, m.p. 156.8°C. Rf = 0.4 Chloroform/Methanol (9.5:0.5) ^1^H‐NMR (400 MHz CDCl_3_) *δ* = 7.74 (d, 1H, J = 8,0, Ar‐H), 7.64 (d, 1H, *J* = 8.0 Hz, Ar‐H), 7.35 (dt, 1H, *J* = 7.4, 1.6 Hz, Ar‐H), 7.32–7.26 (m, 3H, Ar‐H), 7.24 (dt, 1H, *J* = 7.4, 1.6 Hz, Ar‐ H), 7.21–7.16 (m, 2H, Ar‐H), 3.78 ppm (s, 2H, –CH_2_–). ^13^C‐NMR (100 MHz CDCl_3_) *δ* = 169.28, 158.22, 147.94, 132.65, 132.10, 129.52, 129.42, 128.19, 128.13, 126.39, 124.16, 121.52, 120.74, 43.62 ppm. IR (cm^−1^): 3200, 3029, 2969, 1738, 1542, 1495, 1453, 1440, 1414, 1365, 1353, 1228, 1216. HRMS: *m/z* [M+H]^+^ calcd. For C_15_H_12_N_2_OS: 269.0749, Found: 269.0733.

*N*‐(Benzo[*d*]thiazol‐2‐yl)‐2‐(2,5‐dimethoxyphenyl) acetamide (**20**): Orange crystals. Yield: 28.1%, m.p. 152.7°C. Rf = 0.75 Chloroform/Methanol (9.5:0.5) ^1^H‐NMR (400 MHz CDCl_3_) *δ* = 7.83(d, 1H, *J* = 8.0 Hz, Ar‐H), 7.76 (d, 1H, *J* = 8.0 Hz, Ar‐H), 7.44(t, 1H, J = 7.7 Hz, Ar‐H), 7.32(t, 1H, *J* = 7.7 Hz, Ar‐H), 6.94–6.86 (m, 3H, ABX system, Ar‐H), 3.93 (s, 3H, –OCH_3_), 3.85 (s, 2H, –CH2–), 3.81 ppm (s, 3H, –OCH_3_). ^13^C‐NMR (100 MHz CDCl_3_) *δ* = 169.31, 157.94, 154.14, 151.13, 148.21, 132.23, 126.21, 123.89, 122.38, 121.39, 120.76, 117.30, 114.17, 112.28, 56.34, 55.78, 39.21 ppm. IR (cm^−1^): 3450, 3015, 2969, 2946, 2813, 1739, 1549, 1496, 1444, 1365, 1268, 1216, 1109. HRMS: *m/z* [M+H]^+^ calcd. For C_17_H_16_N_2_O_3_S: 329.0959, Found: 329.0947.

*N*‐(Benzo[*d*]thiazol‐2‐yl)‐2‐(3‐methoxyphenyl) acetamide (**21**): Yellow crystals, m.p.: 245.5°C, Yield: 80.56% Rf = 0.65 Chloroform/Methanol (9.5:0.5) ^1^H NMR (400 MHz, CDCl_3_) *δ* = 9.87 (s, 1H,–NHCO–), 7.76–7.72 (m, 1H, Ar‐H), 7.64 (d, *J* = 8.0 Hz, 1H, Ar‐H), 7.34 (ddd, *J* = 8.2, 7.3, 1.2 Hz, 1H, Ar‐H), 7.23 (ddd, *J* = 8.2, 7.3, 1.2 Hz, 1H, Ar‐H), 7.18 (t, J = 8.0 Hz, 1H, Ar‐H), 6.80–6.68 (m, 3H, Ar‐H), 3.74 (s, 2H,–CH_2_–), 3.69 ppm (s, 3H, –OCH_3_). ^13^C‐NMR (100 MHz CDCl_3_) *δ* = 169.24, 160.32, 158.30, 147.97, 134.07, 132.11, 130.46, 126.38, 124.14,121.68, 121.52, 120.73, 115.26, 113.56, 55.25, 43.62 ppm. IR (cm^−1^): 3252, 3202, 3057, 2970, 2831, 1738, 1701, 1595, 1544, 1490, 1466, 1451, 1434, 1416, 1231, 1217, 1151, 1082. HRMS: *m/z* [M+H]^+^ calcd. For C_16_H_14_N_2_O_2_S: 299.0854, Found: 299.0839.

*N*‐(Benzo[*d*]thiazol‐2‐yl)‐2‐(4‐fluorophenyl) acetamide (**22**): Yellow crystals. Yield: 54.10%, m.p. 171.4°C. Rf = 0.54 Chloroform/Methanol (9.5:0.5) ^1^H‐NMR (400 MHz CDCl_3_) *δ* = 7.76 (d, 1H, *J* = 8.5 Hz, Ar‐H), 7.66 (d, 1H, *J* = 8.5 Hz, Ar‐H), 7.36 (t, 1H, *J* = 8.5, 0.7 Hz, Ar‐H), 7.25 (t, 1H, *J* = 8.5, 0.7 Hz, Ar‐H), 7.13 (quasi dd, 2H, AA’ part of AA'BB’ system, *J* = 8.5, 4.2 Hz, Ar‐H), 6.95 (quasi t, 2H, BB’ part of AA'BB’ system, *J* = 8.5, 0.7 Hz, Ar‐H), 3.73 ppm (s, 2H, –CH_2_–). ^13^C‐NMR (100 MHz CDCl_3_) *δ* = 169.09, 163.71, 161.25, 158.36, 147.93, 132.10, 131.14, 128.46, 126.45, 124.24, 121.58, 120.74, 116.33, 116.12, 42.61 ppm. IR (cm^−1^): 3200, 2969, 1738, 1698, 1548, 1508, 1454, 1440, 1422, 1410, 1355, 1217, 1150, 1089, 756. HRMS: *m/z* [M+H]^+^ calcd. For C_15_H_11_FN_2_OS: 287.0654, Found: 287.0640.

*N*‐(Benzo[*d*]thiazol‐2‐yl)‐2‐(4‐chlorophenyl) acetamide (**23**): White crystals, m.p.: 202.3°C, yield: 90.46%. Rf = 0.57 Chloroform/Methanol (9.5:0.5) ^1^H NMR (400 MHz, DMSO‐d_6_) δ = 12.65 (s, 1H, –NHCO–), 7.98 (dd, *J* = 7.8, 0.6 Hz, 1H, Ar‐H), 7.77 (d, J = 7.8 Hz, 1H, Ar‐H), 7.48–7.37 (m, 5H, Ar‐H), 7.34–7.29 (m, 1H, Ar‐H), 3.87 ppm (s, 2H, −CH_2_). ^13^C NMR (100 MHz, DMSO‐d_6_) *δ* = 169.85, 157.84, 148.51, 133.63, 131.68, 131.45, 131.26, 128.34, 126.09, 123.55, 121.67, 120.54, 41.04 ppm. IR (cm^−1^): 3150, 3050, 2965, 1738, 1703, 1690, 1544, 1453, 1440, 1409, 1351, 1282, 1262, 1230, 1216, 1087. HRMS: *m/z* [M+H]^+^ calcd. For C_15_H_11_ClN_2_OS: 303.0359, Found: 303.0346.

*N*‐(Benzo[*d*]thiazol‐2‐yl)‐2‐(4‐methoxyphenyl) acetamide (**24**): Off‐white crystals. Yield: 87.59%. m.p.: 232.7°C Rf = 0.57 Chloroform/Methanol (9.5:0.5) ^1^H NMR (400 MHz, CDCl_3_) *δ* = 7.74 (dd, *J* = 8.1, 0.6 Hz, 1H, Ar‐H), 7.66–7.62 (m, 1H, Ar‐H), 7.34 (ddd, *J* = 8.1, 7.3, 1.3 Hz, 1H, Ar‐H), 7.27–7.20 (m, 1H, Ar‐H), 7.14–7.07 (m, 2H, Ar‐H), 6.84–6.77 (m, 2H, Ar‐H), 3.73 (s, 3H, –OCH_3_), 3.72 ppm (s, 2H, –CH_2_–). ^13^C NMR (100 MHz, CDCl_3_) *δ* = 169.66, 159.55, 158.03, 147.96, 132.09, 130.74, 126.35, 124.46, 124.12, 121.48, 120.76, 114.97, 55.37, 42.79 ppm. IR (cm^−1^): 3150, 3015, 2969, 2945, 2800, 1738, 1698, 1541, 1510, 1454, 1439, 1412, 1365, 1229, 1216, 1151, 761. HRMS: *m/z* [M+H]^+^ calcd. For C_16_H_14_N_2_O_2_S: 299.0854, Found: 299.0838.

*N*‐(Benzo[*d*]thiazol‐2‐yl)‐2‐(thiophen‐2‐yl) acetamide (**25**): Brown Crystals, m.p.: 235°C, Yield: 69.45%. Rf = 0.65 Chloroform/Methanol (9.5:0.5) ^1^H NMR (400 MHz, CDCl_3_) *δ* = 7.75 (dd, J = 7.8, 1.2 Hz, 1H, Ar‐H), 7.70–7.64 (m, 1H, Ar‐H), 7.40–7.32 (m, 1H, Ar‐H), 7.25 (td, *J* = 7.8, 1.2 Hz, 1H, Ar‐H), 7.17 (dd, *J* = 4.1, 1.2 Hz, 1H, Ar‐H), 6.89 (dd, *J* = 4.1, 3.4 Hz, 1H, Ar‐H), 6.85 (dd, J = 3.4, 1.2 Hz, 1H, Ar‐H), 3.97 ppm (s, 2H, –CH_2_–). ^13^C NMR (100 MHz, CDCl_3_) *δ* = 168.28, 158.44, 148.05, 133.64, 132.18, 128.11, 127.64, 126.45, 126.40, 124.23, 121.59, 120.76, 37.40 ppm. IR (cm^−1^): 3450, 3015, 2969, 2946, 1739, 1552, 1454, 1444, 1365, 1269, 1228, 1216, 1174, 753. HRMS: *m/z* [M+H]^+^ calcd. For C_13_H_10_N_2_OS_2_: 275.0312, Found: 275.0298.

#### 4.1.4 General Synthesis Method for Compounds 10–18, 26–28

2‐Aminomethylbenzothiazole (1.1 mmol) was dissolved in 15 mL of dichloromethane and stirred for 20 min. Then, benzoyl chloride or its derivatives, 2‐furoyl chloride, 2‐thiophenecarbonyl chloride, phenylacetyl chloride, or its derivatives, or 2‐thiopheneacetyl chloride (1 mmol) was added to the solution in an ice bath. The mixture was stirred at room temperature for 2 h. Subsequently, 10 drops of DIPEA were added to the solution, and the reaction mixture was stirred at room temperature for 48 h. The completion of the reaction was monitored by TLC. After completion, the reaction was quenched, and dichloromethane (45 mL) was added to the solution. The organic phase was washed with 1 N HCl (3 × 20 mL), followed by a saturated NaHCO_3_ solution (2 × 15 mL), and 15 mL of distilled water. The organic phase was separated, dried over MgSO_4_, filtered, and the solvent was evaporated under reduced pressure. The solid product was then purified by column chromatography using an appropriate mobile phase (n‐hexane/ethyl acetate). The final products were recrystallized using an appropriate solvent.

*N*‐(Benzo[*d*]thiazol‐2‐ylmethyl) benzamide (**10**): White crystals, m.p.:202.2°C, Yield: 70.6%. Rf = 0.62 Chloroform/Methanol (9.5:0.5) ^1^H NMR (400 MHz, MeOD) *δ* = 7.85–7.83 (m, 1H, Ar‐H), 7.82 (dd, *J* = 1.7, 0.9 Hz, 2H, Ar‐H), 7.80 (t, J = 1.7 Hz, 1H, Ar‐H), 7.49–7.44 (m, 1H, Ar‐H), 7.42–7.36 (m, 3H, Ar‐H), 7.32–7.27 (m, 1H, Ar‐H), 4.87 (s, 2H, –CH_2_–) ppm. ^13^C NMR (100 MHz, MeOD) *δ* = 172.88, 170.50, 154.09, 136.19, 135.00, 133.16, 129.74, 128.53, 127.44, 126.45, 123.42, 123.04, 43.06 ppm. IR (cm^−1^): 3273, 1637, 1601, 1521, 1484, 1454, 1310, 1292, 1052, 665. HRMS: *m/z* [M+H]^+^ calcd. For C_15_H_12_N_2_OS: 269.0749, Found: 269.0734.

*N*‐(Benzo[*d*]thiazol‐2‐ylmethyl)‐4‐chlorobenzamide (**11**): White crystals, m.p.: 237°C, Yield: 71%. Rf = 0.70 Chloroform/Methanol (9.5:0.5) 1H NMR (400 MHz, DMSO‐d_6_) *δ* = 9.65 (t, *J* = 5.8 Hz, 1H, –NHCO–), 8.07 (ddd, *J* = 7.6, 1.2, 0.6 Hz, 1H, Ar‐H), 8.01–7.93 (m, 3H, Ar‐H), 7.67–7.60 (m, 2H, Ar‐H), 7.55–7.50 (m, 1H, Ar‐H), 7.44 (ddd, *J* = 8.3, 7.6, 1.2 Hz, 1H, Ar‐H), 4.90 ppm (d, *J* = 5.8 Hz, 2H, –CH2–). ^13^C NMR (100 MHz, DMSO‐d6) *δ* = 171.02, 165.69, 152.69, 136.56, 134.56, 132.34, 129.27, 128.61, 126.14, 125.02, 122.37, 122.24, 41.77 ppm. IR (cm^−1^): 3245, 1621, 1594, 1540, 1487, 1447, 1436, 1409, 1316, 1305, 1277, 1160, 1144, 867, 759, 727. HRMS: *m/z* [M+H]^+^ calcd. For C_15_H_11_ClN_2_OS: 303.0359, Found: 303.0345.

*N*‐(Benzo[*d*]thiazol‐2‐ylmethyl)‐4‐fluorobenzamide (**12**): White crystals, m.p.: 235°C, Yield: 84%. Rf = 0.48 Chloroform/Methanol (9.5:0.5) ^1^H NMR (400 MHz, DMSO‐d_6_) *δ* = 9.58 (t, *J* = 5.8 Hz, 1H, –NHCO–), 8.07 (ddd, *J* = 8.1, 1.2, 0.6 Hz, 1H, Ar‐H), 8.05–8.00 (m, 2H, Ar‐H), 8.00–7.96 (m, 1H, Ar‐H), 7.53 (ddd, *J* = 8.1, 7.2, 1.2 Hz, 1H, Ar‐H), 7.46–7.42 (m, 1H, Ar‐H), 7.41–7.35 (m, 2H, Ar‐H), 4.90 ppm (d, *J* = 5.8 Hz, 2H, –CH_2_–). ^13^C NMR (100 MHz, DMSO‐d_6_) *δ* = 171.16, 165.67, 165.38, 162.91, 152.72, 134.57, 130.08, 129.99, 126.12, 125.00, 122.36, 122.22, 115.56, 115.34, 41.77 ppm. IR (cm^−1^): 3234, 1627, 1598, 1551, 1518, 1504, 1456, 1351, 1291, 1258, 861. HRMS: *m/z* [M+H]^+^ calcd. For C_15_H_11_FN_2_OS: 287.0654, Found: 287.0637.

*N*‐(Benzo[*d*]thiazol‐2‐ylmethyl)‐4‐(trifluoromethyl) benzamide (**13**): White to yellow crystals, m.p.: 252°C, Yield: 83%. Rf = 0.38 Chloroform/Methanol (9.5:0.5) ^1^H NMR (400 MHz, DMSO‐d_6_) *δ* = 9.79 (t, *J* = 5.9 Hz, 1H, –NHCO–), 8.15 (d, *J* = 8.1 Hz, 2H, Ar‐H), 8.08 (dd, *J* = 8.1, 0.6 Hz, 1H, Ar‐H), 8.01–7.97 (m, 1H, Ar‐H), 7.94 (d, *J* = 8.1 Hz, 2H, Ar‐H), 7.53 (ddd, *J* = 8.1, 7.3, 1.3 Hz, 1H, Ar‐H), 7.48–7.41 (m, 1H, Ar‐H), 4.93 ppm (d, *J* = 5.9 Hz, 2H, −CH_2_‐). ^13^C NMR (100 MHz, DMSO‐d_6_) δ = 170.70, 165.61, 152.69, 137.38, 134.58, 131.72, 131.40, 128.26, 126.15, 125.56, 125.53, 125.05, 122.40, 122.23, 41.83 ppm. IR (cm^−1^): 3328, 1646, 1540, 1506, 1414, 1326, 1289, 1168, 1069, 1038, 774, 717, 695. HRMS: *m/z* [M+H]^+^ calcd. For C_16_H_11_F_3_N_2_OS: 337.0622, Found: 337.0603.

*N*‐(Benzo[*d*]thiazol‐2‐ylmethyl) thiophene‐2‐carboxamide (**14**): White to yellow crystals, m.p.: 240°C, Yield: 40%. Rf = 0.61 Chloroform/Methanol (9.5:0.5) ^1^H NMR (400 MHz, DMSO‐d_6_) *δ* = 9.55 (t, J = 5.7 Hz, 1H, –NHCO–), 8.08 (d, *J* = 7.9 Hz, 1H, Ar‐H), 7.99 (d, *J* = 7.9 Hz, 1H, Ar‐H), 7.89 (d, J = 3.9 Hz, 1H, Ar‐H), 7.86 (d, *J* = 3.9 Hz, 1H, Ar‐H), 7.53 (dd, *J* = 9.4, 3.9 Hz, 1H, Ar‐H), 7.44 (t, *J* = 9.4 Hz, 1H, Ar‐H), 7.25–7.20 (m, 1H, Ar‐H), 4.88 ppm (d, *J* = 5.7 Hz, 2H, –CH_2_–). 13 C NMR (100 MHz, DMSO‐d_6_) *δ* = 171.00, 161.60, 152.70, 138.90, 134.61, 131.48, 128.82, 128.08, 126.14, 125.02, 122.38, 122.25, 41.55 ppm. IR (cm^−1^): 3357, 1633, 1536, 1518, 1507, 1436, 1416, 1313, 1299, 1235, 1167, 760, 721. HRMS: *m/z* [M+H]^+^ calcd. For C_13_H_10_N_2_OS_2_: 275.0313, Found: 275.0302.

*N*‐(Benzo[*d*]thiazol‐2‐ylmethyl) furan‐2‐carboxamide (**15**): Yellow crystals, m.p.: 224°C, Yield: 51%. Rf = 0.67 Chloroform/Methanol (9.5:0.5) ^1^H NMR (400 MHz, DMSO‐d_6_) *δ* = 9.40 (t, *J* = 6.0 Hz, 1H, –NHCO–), 8.07 (ddd, *J* = 8.0, 1.2, 0.8 Hz, 1H, Ar‐H), 8.02–7.96 (m, 1H, Ar‐H), 7.93 (dd, *J* = 1.7, 0.8 Hz, 1H, Ar‐H), 7.52 (ddd, *J* = 8.2, 7.2, 1.2 Hz, 1H, Ar‐H), 7.44 (ddd, *J* = 8.2, 7.2, 1.2 Hz, 1H, Ar‐H), 7.24 (dd, *J* = 3.5, 0.8 Hz, 1H, Ar‐H), 6.70 (dd, *J* = 3.5, 1.7 Hz, 1H, Ar‐H), 4.85 ppm (d, *J* = 6.0 Hz, 2H, –CH_2_–). ^13^C NMR (100 MHz, DMSO‐d_6_) *δ* = 170.98, 158.12, 152.69, 147.30, 145.54, 134.58, 126.12, 124.99, 122.37, 122.22, 114.25, 111.99, 41.06 ppm. IR (cm^−1^): 3222, 1667, 1648, 1570, 1529, 1472, 1302, 1196, 759, 749, 727. HRMS: *m/z* [M+H]^+^ calcd. For C_13_H_10_N_2_O_2_S: 259.0541, Found: 259.0526.

*N*‐(Benzo[*d*]thiazol‐2‐ylmethyl)‐4‐bromobenzamide (**16**): White crystals, m.p.: 234°C, Yield: 80.7%. Rf = 0.63 Chloroform/Methanol (9.5:0.5)^1^H NMR (400 MHz, DMSO‐d_6_) *δ* = 9.63 (t, *J* = 5.9 Hz, 1H, –NHCO–), 8.09–8.06 (m, 1H, Ar‐H), 7.98 (dd, *J* = 8.2, 0.5 Hz, 1H, Ar‐H), 7.91 (t, J = 2.2 Hz, 1H, Ar‐H), 7.89–7.88 (m, 1H, Ar‐H), 7.79–7.77 (m, 1H, Ar‐H), 7.77–7.74 (m, 1H, Ar‐H), 7.53 (ddd, *J* = 8.2, 7.2, 1.2 Hz, 1H, Ar‐H), 7.44 (ddd, J = 8.2, 7.2, 1.2 Hz, 1H, Ar‐H), 4.90 ppm (d, J = 5.9 Hz, 2H, –CH2–). ^13^C NMR (100 MHz, DMSO‐d6) *δ* = 170.96, 165.83, 152.70, 134.58, 132.75, 131.54, 129.45, 126.13, 125.49, 125.01, 122.37, 122.23, 41.77 ppm. IR (cm^−1^): 3278, 1629, 1589, 1540, 1479, 1435, 1410, 1345, 1273, 1163, 756, 725, 624. HRMS: *m/z* [M+H]^+^ calcd. For C_15_H_11_BrN_2_OS: 346.9854, Found: 346.9839.

*N*‐(Benzo[*d*]thiazol‐2‐ylmethyl)‐4‐methoxybenzamide (**17**): Light brown crystals, m.p.: 218°C, Yield: 93.6%. Rf = 0.73 Chloroform/Methanol (9.5:0.5)^1^H NMR (400 MHz, DMSO‐d_6_) *δ* = 9.40 (t, *J* = 5.9 Hz, 1H, –NHCO–), 8.08–8.05 (m, 1H, Ar‐H), 8.00–7.97 (m, 1H, Ar‐H), 7.96–7.95 (m, 1H, Ar‐H), 7.94–7.92 (m, 1H, Ar‐H), 7.52 (ddd, *J* = 8.3, 7.3, 1.3 Hz, 1H, Ar‐H), 7.46–7.41 (m, 1H, Ar‐H), 7.09–7.05 (m, 2H, Ar‐H), 4.88 (d, *J* = 5.9 Hz, 2H, –CH_2_–), 3.85 ppm (s, 3H, –OCH3). ^13^C NMR (100 MHz, DMSO‐d_6_) *δ* = 171.65, 166.19, 161.94, 152.75, 134.59, 129.23, 126.08, 125.84, 124.94, 122.33, 122.20, 113.70, 55.40, 41.70 ppm. IR (cm^−1^): 3294, 1637, 1604, 1548, 1516, 1505, 1407, 1236, 1188, 1161, 1028, 758, 697. HRMS: *m/z* [M+H]^+^ calcd. For C_16_H_14_N_2_O_2_S: 299.0854, Found: 299.0843.

*N*‐(Benzo[*d*]thiazol‐2‐ylmethyl)‐4‐methylbenzamide (**18**): Light brown crystals, m.p.: 218°C, Yield: 93.6%. Rf = 0.75 Chloroform/Methanol (9.5:0.5) ^1^H NMR (400 MHz, DMSO‐d_6_) *δ* = 9.46 (t, *J* = 5.9 Hz, 1H, –NHCO–), 8.06 (ddd, *J* = 8.1, 1.2, 0.6 Hz, 1H, Ar‐H), 8.00–7.95 (m, 1H, Ar‐H), 7.91–7.84 (m, 2H, Ar‐H), 7.52 (ddd, *J* = 8.1, 7.2, 1.2 Hz, 1H, Ar‐H), 7.45–7.40 (m, 1H, Ar‐H), 7.34 (d, *J* = 8.1 Hz, 2H, Ar‐H), 4.88 (d, *J* = 5.9 Hz, 2H, –CH_2_–), 2.39 ppm (s, 3H, –CH_3_). ^13^C NMR (100 MHz, DMSO‐d6) *δ* = 171.48, 166.62, 152.74, 141.65, 134.58, 130.87, 128.99, 127.36, 126.09, 124.95, 122.34, 122.20, 41.71, 20.97 ppm. IR (cm^−1^): 3278, 1638, 1608, 1539, 1501, 1454, 1437, 1315, 1238, 1150, 756, 298, 669. HRMS: *m/z* [M+H]^+^ calcd. For C_16_H_14_N_2_OS: 283.0905, Found: 283.0891.

*N*‐(Benzo[*d*]thiazol‐2‐ylmethyl)‐2‐(3‐methoxyphenyl) acetamide (**26**): White yellow crystals, m.p.: 234.8°C, Yield: 15%. Rf = 0.47 Chloroform/Methanol (9.5:0.5)^1^H NMR (400 MHz, MeOD) δ = 7.82 (ddd, *J* = 3.1, 1.2, 0.6 Hz, 1H, Ar‐H), 7.81–7.78 (m, 1H, Ar‐H), 7.38 (ddd, *J* = 8.3, 7.3, 1.2 Hz, 1H, Ar‐H), 7.30 (ddd, *J* = 8.3, 7.3, 1.2 Hz, 1H, Ar‐H), 7.16–7.10 (m, 1H, Ar‐H), 6.84–6.79 (m, 2H, Ar‐H), 6.74–6.70 (m, 1H, Ar‐H), 4.67 (s, 2H, –NCH_2_–) 3.67 (s, 3H, –OCH_3_), 3.50 ppm (s, 2H, –COCH_2_–). ^13^C NMR (100 MHz, MeOD) *δ* = 174.37, 172.42, 161.41, 154.00, 137.84, 136.19, 130.65, 127.43, 126.47, 123.40, 122.99, 122.60, 115.88, 113.74, 55.70, 43.80, 42.68 ppm. IR (cm^−1^): 3450, 3303, 3004, 2969, 2928, 2848, 1739, 1658, 1608, 1584, 1534, 1508, 1491, 1433, 1365, 1216, 760. HRMS: *m/z* [M+H]^+^ calcd. For C_17_H_16_N_2_O_2_S: 313.1011, Found: 313.0995.

N‐(Benzo[*d*]thiazol‐2‐ylmethyl)‐2‐(4‐chlorophenyl) acetamide (**27**): White crystals, m.p.: 250.4°C, Yield: 15%. Rf = 0.65 Chloroform/Methanol (9.5:0.5)^1^H NMR (400 MHz, MeOD) *δ* = 7.84–7.79 (m, 2H, Ar‐H), 7.39 (ddd, *J* = 8.3, 7.3, 1.1 Hz, 1H, Ar‐H), 7.30 (ddd, J = 8.3, 7.3, 1.1 Hz, 1H, Ar‐H), 7.22 (m, 4H, Ar‐H), 4.67 (s, 2H, –NCH_2_–), 3.52 ppm (s, 2H, –COCH_2_–). ^13^C NMR (100 MHz, MeOD) *δ* = 173.91, 172.28, 154.00, 136.18, 135.30, 134.01, 131.93, 129.68, 127.45, 126.49, 123.41, 123.02, 42.90, 42.68 ppm. IR (cm^−1^): 3450, 3264, 3015, 2969, 2946, 1739, 1648, 1544, 1515, 1488, 1436, 1365, 1365, 1216, 1090, 755. HRMS: *m/z* [M+H]^+^ calcd. For C_16_H_13_ClN_2_OS: 317.0515, Found: 317.0511.

*N*‐(Benzo[*d*]thiazol‐2‐ylmethyl)‐2‐(thiophen‐2‐yl) acetamide (**28**): White crystals, m.p.: 237.1°C, Yield: 30%. Rf = 0.52 Chloroform/Methanol (9.5:0.5)^1^H NMR (400 MHz, MeOD) *δ* = 7.82 (dd, *J* = 2.8, 1.0 Hz, 1H, Ar‐H), 7.80–7.78 (m, 1H, Ar‐H), 7.40–7.35 (m, 1H Ar‐H), 7.32–7.26 (m, 1H, Ar‐H), 7.18 (dd, *J* = 5.1, 1.0 Hz, 1H, Ar‐H), 6.90 (d, *J* = 3.4 Hz, 1H, Ar‐H), 6.86 (dd, *J* = 5.1, 3.4 Hz, 1H, Ar‐H), 4.67 (s, 2H, –NCH_2_–), 3.74 ppm (s, 2H,–COCH_2_–). ^13^C NMR (100 MHz, MeOD) *δ* = 173.28, 172.27, 154.00, 137.51, 136.22, 127.96, 127.92, 127.43, 126.48, 126.01, 123.41, 123.02, 42.73, 37.73 ppm. IR (cm^−1^): 3450, 3291, 3015, 2969, 2946, 1739, 1655, 1540, 1507, 1454, 1433, 1425, 1365, 1229, 1216, 752. HRMS: *m/z* [M+H]^+^ calcd. For C_14_H_12_N_2_OS_2_: 289.0469, Found: 289.0455.

### 4.2 Biological Assays

#### 4.2.1 Cell Culture

Human epithelial breast adenocarcinoma (MDA‐MB‐231) (ATCC® HTB‐26™), human epithelial breast adenocarcinoma (MCF7) (ATCC® HTB‐22™), and healthy mouse fibroblast (L929) (ATCC® CRL‐10317™) cells were obtained commercially from ATCC.

#### 4.2.2 Cells Proliferation

MDA‐MB‐231, MCF7, and L929 cells were seeded in 25 cm² flasks. DMEM medium containing 10% FBS, 1% Penicillin‐Streptomycin, l‐Glutamine, and NaHCO₃ was used as the cell culture medium. The cells were incubated and propagated at 37°C in an environment containing 5% CO₂ and 95% humidity. When the cells reached 80%–90% confluency, they were passaged to different media. During passaging, DMEM was first withdrawn, and the cells were washed with PBS to remove FBS from the medium. Then, Trypsin‐EDTA solution was added to the flask to detach the cells, and the flask was kept in a CO₂ incubator for approximately 1 min. To stop the activity of Trypsin‐EDTA medium containing FBS was added to the flask. The cells were collected into a Falcon tube and centrifuged at 800 rpm for 5 min at room temperature. The supernatant was discarded, and the cell pellet was resuspended in the medium and seeded on cell culture plates according to the experimental protocol.

##### 4.2.2.1 Cell Counting

To ensure a consistent number of cells in each well before plating, cells were stained with Trypan Blue after passage and counted using an Olympus R1 device. After mixing the cells with Trypan Blue and incubating at room temperature for 5 min, 10 μL was loaded into the counting slide and analyzed. Viable cells were counted by identifying only the unstained cells. The number of cells to be plated in each well was determined by calculating the number of cells per mL, considering the dilution factor.

##### 4.2.2.2 Cytotoxic Activities

A total of 2 mg of each compound was dissolved in an appropriate amount of dimethyl sulfoxide (DMSO) to prepare 10 mM stock solutions. These stock solutions were then diluted to create 100 μM intermediate stocks and stored at −80°C. Commercially purchased cisplatin and doxorubicin HCl (Sigma, St. Louis, MO) were dissolved in DMSO following the manufacturer's protocol to create 20 mM stock solutions. These standard drug molecules were diluted from the stock solution, divided into 100 μM aliquots, and stored at –80°C until use.

MTT analysis was performed to evaluate the in vitro cytotoxicity of the synthesized compounds against different cancer cell lines. Cells used in the cytotoxicity experiments were cultured in DMEM medium supplemented with 10% FBS. Cells suspended in the medium (2 × 10⁴ cells/mL) were seeded into 96‐well culture plates and incubated at 37°C in a 5% CO₂ incubator. After 12 h, compounds were added at different doses (2 µL) to cells (2 × 10⁴) in the 96‐well plates and incubated at 37°C for 24 h.

The incubated cells were then treated with 20 µL of MTT solution and incubated at 37°C for 4 h. The supernatant was carefully removed from each well, and 100 µL of DMSO was added to each well to dissolve the formazan crystals formed by the cellular reduction of MTT. The absorbance was measured at 570 nm using an ELISA reader. The results are expressed as IC_50_ (µM), which indicates the concentration required to inhibit 50% of cell growth compared with control cells. At the end of the experiment, the device calculated the IC_50_ value for each well, and the average values were recorded. Each experiment was performed in at least three replicates.

#### 4.2.3 Total Protein Determination (BCA Protein Assay) [52]

A bicinchoninic acid (BCA) protein assay kit (catalog no. 23227) was obtained from Thermo‐Fisher Scientific (IL, USA) and used to detect total protein by the BCA assay. In this assay, the reaction between BCA, Cu²⁺ ions, and Cu⁺ ions form a purple complex in alkaline conditions. The absorbance of this complex was measured at 562 nm, and the results were expressed in mg/mL.

#### 4.2.4 p38 MAPK Alpha Assay [53]

The p38 MAPK alpha ELISA Kit (ab221012; Abcam, Cambridge, MA) was used to measure the levels of p‐MAPK in cell lysates according to the manufacturer's instructions. The SimpleStep Enzyme‐Linked Immunosorbent Assay (ELISA) kit was designed to assess total p38 MAPKα protein semi‐quantitatively. The SimpleStep ELISA uses a reporter‐conjugated detection antibody, and an affinity‐tag labeled capture antibody to immunocapture the sample analyte in solution. An anti‐tag antibody coating the well immobilizes the entire complex (capture antibody, analyte, and detection antibody).

Samples or controls were added to the wells, followed by the Antibody Cocktail to complete the test. After incubation, the wells were washed to remove unbound material. The addition of TMB substrate resulted in a blue color formation catalyzed by HRP during incubation. The levels of MAPK were normalized to total protein content.

### 4.3 In Silico Studies

#### 4.3.1 Molecular Docking

All molecular docking studies were carried out using Maestro 12.4 (Schrodinger). Crystal structures of p38α MAPK (PDB: 1A9U and 4DLI) were downloaded from the RCSB Protein Data Bank (www.rcsb.org) and prepared with the Protein Preparation module. Water molecules in the crystal structure were removed, hydrogen atoms were added, bond orders were adjusted, and energy minimization was carried out using the OPLS3e force field. The proteins were minimized accordingly. A grid map with 20 Å on each side was created in the active regions of the proteins.

Benzothiazole derivatives were drawn using the 2D Sketcher module of the Maestro program; 3D structures were prepared with LigPrep software for molecular modeling studies. The ligands were docked into the active sites of the 1A9U and 4DLI pdb‐encoded proteins 100 times at standard precision (SP) using Glide, and the best poses were used in molecular dynamics simulations [29, 54, 55].

#### 4.3.2 MM/GBSA Calculations

The binding energy was computed by Prime MM/GBSA in Schrödinger Suite 12.4. MM/GBSA uses OPLS‐3A molecular mechanics energies, a VSGB solvation model for polar solvation (G_SGB_), and a nonpolar solvation expression (G_NP_) that includes van der Waals interactions and nonpolar solvent‐accessible surface area (SASA). Binding energies of ligands were calculated as kcal/mol (Table 4) [56, 57].

#### 4.3.3 Molecular Dynamic Simulation

The Desmond program (Schrodinger, 12.4) was employed for all molecular dynamics' simulations. Simulations were initiated with the best pose obtained from the molecular docking studies. 100 ns molecular dynamics simulations were conducted using the NPT ensemble for compounds 15 and 19 (the most potent compounds) against the 1A9U and 4DLI proteins. In these simulations, RMSD values of the ligands and proteins, as well as ligand–protein interactions, were evaluated. The ligands were immersed in a solvent by placing them in an octahedral box with TIP3P water molecules, ensuring a minimum distance of 10 Å between the ligands and the box edges. The systems were rendered chemically neutral by adding Na⁺ and Cl⁻ ions, and the ionic concentration of the systems was adjusted using a 0.15 M NaCl solution. Desmond's standard relaxation protocol was employed. The Nose–Hoover chain algorithm was used to maintain the temperature at 300 K, and the Martyna–Tobias–Klein algorithm was applied to regulate the pressure at 1.01325 bar [58, 59, 60].

## Conflicts of Interest

The authors declare no conflicts of interest.

## Supporting information

ArchPharm_SupplMat_InChI.doc.

Supporting_Information.doc.

## Data Availability

The authors have nothing to report.
