# Supplementary material for: Development of Potent Type V MAPK Inhibitors: Design, Synthesis, and Biological Evaluation of Benzothiazole Derivatives Targeting p38α MAPK in Breast Cancer Cells
Source: Arch Pharm (Weinheim). 2025 Apr 7;358(4):e2500011. doi: 10.1002/ardp.202500011 (PMC11975549; doi:10.1002/ardp.202500011)
Supplement: Supplementary file 1 — ArchPharm_SupplMat_InChI.doc. [file ARDP-358-e2500011-s002.doc]

**Supplemental Material: Novel Compounds and Biological Screening Results**

**Synthesis, biological evaluation, and in silico studies of benzothiazole derivatives as potential p38α MAPK inhibitors targeting human breast cancer MCF-7 cells**

Bayan Zoatier^1^, K. Gizem Yildiztekin^2^, M. Abdullah Alagoz^3^, Ceylan Hepokur^4^, Serdar Burmaoglu^5^, Oztekin Algul^1^, *

^1^Department of Pharmaceutical Chemistry, Faculty of Pharmacy, Mersin University, Mersin, Türkiye

^2^Department of Toxicology, Faculty of Pharmacy, Erzincan Binali Yıldırım University, Erzincan, Türkiye

^3^Department of Pharmaceutical Chemistry, Faculty of Pharmacy, Inonu University, Malatya, Türkiye

^4^Department of Biochemistry, Faculty of Pharmacy, Sivas Cumhuriyet University, Sivas, Türkiye

^5^Department of Chemistry, Faculty of Science, Atatürk University, Erzurum, Türkiye

*Correspondence:

Prof., Oztekin Algul, Department of Pharmaceutical Chemistry, Faculty of Pharmacy, Yenisehir Campus C Block, 33160, Mersin Türkiye

Email: oztekinalgul@mersin.edu.tr

| **Compound No.** | **InChI** | **Biological Activity (**Total p38α MAPK levels (ng/μg protein)**)^a^**  **(**MCF7 IC_50_ (µM**)^b^** |
| --- | --- | --- |
| 1 | 1S/C15H12N2O2S/c1-19-11-8-6-10(7-9-11)14(18)17-15-16-12-4-2-3-5-13(12)20-15/h2-9H,1H3,(H,16,17,18) | 7.367 ± 1.291 |
| 2 | 1S/C14H10N2O2S/c17-10-7-5-9(6-8-10)13(18)16-14-15-11-3-1-2-4-12(11)19-14/h1-8,17H,(H,15,16,18) | 3.879 ± 0.868 |
| 3 | 1S/C12H8N2OS2/c15-11(10-6-3-7-16-10)14-12-13-8-4-1-2-5-9(8)17-12/h1-7H,(H,13,14,15) | 0.43 ± 0.005 |
| 4 | 1S/C12H8N2OS2/c15-11(10-6-3-7-16-10)14-12-13-8-4-1-2-5-9(8)17-12/h1-7H,(H,13,14,15) | 8.529 ± 0.9 |
| 5 | 1S/C15H12N2OS/c1-10-6-8-11(9-7-10)14(18)17-15-16-12-4-2-3-5-13(12)19-15/h2-9H,1H3,(H,16,17,18) | 12.93±0.37 |
| 6 | 1S/C15H9F3N2OS/c16-15(17,18)10-7-5-9(6-8-10)13(21)20-14-19-11-3-1-2-4-12(11)22-14/h1-8H,(H,19,20,21) | 13.49±0.37 |
| 7 | 1S/C14H10N2OS/c17-13(10-6-2-1-3-7-10)16-14-15-11-8-4-5-9-12(11)18-14/h1-9H,(H,15,16,17) | 12.39±0.97 |
| 8 | 1S/C15H12N2OS/c1-10-6-8-11(9-7-10)14(18)17-15-16-12-4-2-3-5-13(12)19-15/h2-9H,1H3,(H,16,17,18) | 9.541 ± 0.099 |
| 9 | 1S/C14H9ClN2OS/c15-10-7-5-9(6-8-10)13(18)17-14-16-11-3-1-2-4-12(11)19-14/h1-8H,(H,16,17,18) | 0.166 ± 0.015 |
| 10 | 1S/C15H12N2OS/c18-15(11-6-2-1-3-7-11)16-10-14-17-12-8-4-5-9-13(12)19-14/h1-9H,10H2,(H,16,18) | 0.535 ± 0.079 |
| 11 | 1S/C15H11ClN2OS/c16-11-7-5-10(6-8-11)15(19)17-9-14-18-12-3-1-2-4-13(12)20-14/h1-8H,9H2,(H,17,19) | 15.57 ±0.65 |
| 12 | 1S/C15H11FN2OS/c16-11-7-5-10(6-8-11)15(19)17-9-14-18-12-3-1-2-4-13(12)20-14/h1-8H,9H2,(H,17,19) | 21.09±0.46 |
| 13 | 1S/C16H11F3N2OS/c17-16(18,19)11-7-5-10(6-8-11)15(22)20-9-14-21-12-3-1-2-4-13(12)23-14/h1-8H,9H2,(H,20,22) | 12.93±0.86 |
| 14 | 1S/C13H10N2O2S/c16-13(10-5-3-7-17-10)14-8-12-15-9-4-1-2-6-11(9)18-12/h1-7H,8H2,(H,14,16) | 0.199 ± 0.011 |
| 15 | 1S/C13H10N2OS2/c16-13(11-6-3-7-17-11)14-8-12-15-9-4-1-2-5-10(9)18-12/h1-7H,8H2,(H,14,16) | 0.155 ± 0.045 |
| 16 | 1S/C15H11BrN2OS/c16-11-7-5-10(6-8-11)15(19)17-9-14-18-12-3-1-2-4-13(12)20-14/h1-8H,9H2,(H,17,19) | 14.10±0.95 |
| 17 | 1S/C16H14N2O2S/c1-20-12-8-6-11(7-9-12)16(19)17-10-15-18-13-4-2-3-5-14(13)21-15/h2-9H,10H2,1H3,(H,17,19) | 6.411 ± 1.604 |
| 18 | 1S/C15H11BrN2OS/c16-11-7-5-10(6-8-11)15(19)17-9-14-18-12-3-1-2-4-13(12)20-14/h1-8H,9H2,(H,17,19) | 1.34 ± 0.154 |
| 19 | 1S/C15H12N2OS/c18-14(10-11-6-2-1-3-7-11)17-15-16-12-8-4-5-9-13(12)19-15/h1-9H,10H2,(H,16,17,18) | 0.112 ± 0.001 |
| 20 | 1S/C17H16N2O3S/c1-21-12-7-8-14(22-2)11(9-12)10-16(20)19-17-18-13-5-3-4-6-15(13)23-17/h3-9H,10H2,1-2H3,(H,18,19,20) | 14.69 ±0.15 |
| 21 | 1S/C16H14N2O2S/c1-20-12-6-4-5-11(9-12)10-15(19)18-16-17-13-7-2-3-8-14(13)21-16/h2-9H,10H2,1H3,(H,17,18,19) | 1.333 ± 0.007 |
| 22 | 1S/C15H11FN2OS/c16-11-7-5-10(6-8-11)9-14(19)18-15-17-12-3-1-2-4-13(12)20-15/h1-8H,9H2,(H,17,18,19) | 3.952 ± 0.311 |
| 23 | 1S/C15H11ClN2OS/c16-11-7-5-10(6-8-11)9-14(19)18-15-17-12-3-1-2-4-13(12)20-15/h1-8H,9H2,(H,17,18,19) | 2.331 ± 1.241 |
| 24 | 1S/C16H14N2O2S/c1-20-12-8-6-11(7-9-12)10-15(19)18-16-17-13-4-2-3-5-14(13)21-16/h2-9H,10H2,1H3,(H,17,18,19) | 12.65 ±0.18 |
| 25 | 1S/C13H12N2OS2/c1-2-5-9(17)8-12(16)15-13-14-10-6-3-4-7-11(10)18-13/h2-7,17H,1,8H2,(H,14,15,16)/b9-5- | 13.21 ±0.03 |
| 26 | 1S/C17H16N2O2S/c1-21-13-6-4-5-12(9-13)10-16(20)18-11-17-19-14-7-2-3-8-15(14)22-17/h2-9H,10-11H2,1H3,(H,18,20) | 13.52±0.35 |
| 27 | 1S/C16H13ClN2OS/c17-12-7-5-11(6-8-12)9-15(20)18-10-16-19-13-3-1-2-4-14(13)21-16/h1-8H,9-10H2,(H,18,20) | 12.34±0.96 |
| 28 | 1S/C14H12N2OS2/c17-13(8-10-4-3-7-18-10)15-9-14-16-11-5-1-2-6-12(11)19-14/h1-7H,8-9H2,(H,15,17) | 2.542 ± 0.228 |
