# Supplementary material for: Development of Potent Type V MAPK Inhibitors: Design, Synthesis, and Biological Evaluation of Benzothiazole Derivatives Targeting p38α MAPK in Breast Cancer Cells
Source: Arch Pharm (Weinheim). 2025 Apr 7;358(4):e2500011. doi: 10.1002/ardp.202500011 (PMC11975549; doi:10.1002/ardp.202500011)
Supplement: Supplementary file 2 — Supporting_Information.doc. [file ARDP-358-e2500011-s001.doc]

**SUPPORTING INFORMATION**

**Development of Potent Type V MAPK Inhibitors: Design, Synthesis, and Biological Evaluation of Benzothiazole Derivatives Targeting p38α MAPK in Breast Cancer Cells**

Bayan Zoatier^1^, K. Gizem Yildiztekin^2^, M. Abdullah Alagoz^3^, Ceylan Hepokur^4^, Serdar Burmaoglu^5^, Oztekin Algul^1,6^, *

^1^Department of Pharmaceutical Chemistry, Faculty of Pharmacy, Mersin University, Mersin, Türkiye

^2^Department of Toxicology, Faculty of Pharmacy, Erzincan Binali Yıldırım University, Erzincan, Türkiye

^3^Department of Pharmaceutical Chemistry, Faculty of Pharmacy, Inonu University, Malatya, Türkiye

^4^Department of Biochemistry, Faculty of Pharmacy, Sivas Cumhuriyet University, Sivas, Türkiye

^5^Department of Chemistry, Faculty of Science, Atatürk University, Erzurum, Türkiye

^6^Department of Pharmaceutical Chemistry, Faculty of Pharmacy, Erzincan Binali Yıldırım University, Erzincan, Türkiye

**Contents**

Copies of ^1^H NMR,^13^C NMR, IR and Mass spectra of all compounds………………..……………...2

**Table S1**. The crystal structures contain the known ligands (positive controls) and docking scores against 4DLI………………………………………………………………………………….……..114

**Table S2**. The crystal structures contain the known ligands (positive controls) and docking scores against 1A9U………………………………………………………………………………….…….115

**Figure S1.** Radius of gyration (rGyr) analysis of the ligands in 4DLI and 1A9U during the 100 ns MD simulation………………………………………………………………………………...…………117 **Figure S2.** Solvent Accessible Surface Area (SASA) analysis of the ligands in 4DLI and 1A9U during the 100 ns MD simulation……………………………...……………………………………118

Corresponding author: Oztekin Algul, PhD., Mersin University, Faculty of Pharmacy, Department of Pharmaceutical Chemistry 33169, Mersin, Turkey, Phone: +90 324 3412815/12122; Fax: +90-324 3413022; E-mail: [oztekinalgul@mersin.edu.tr](mailto:oztekinalgul@mersin.edu.tr)

^1^H NMR spectrum of N-(benzo[*d*]thiazol-2-yl)-4-methoxybenzamide (1)

^13^C NMR spectrum of N-(benzo[*d*]thiazol-2-yl)-4-methoxybenzamide (1)


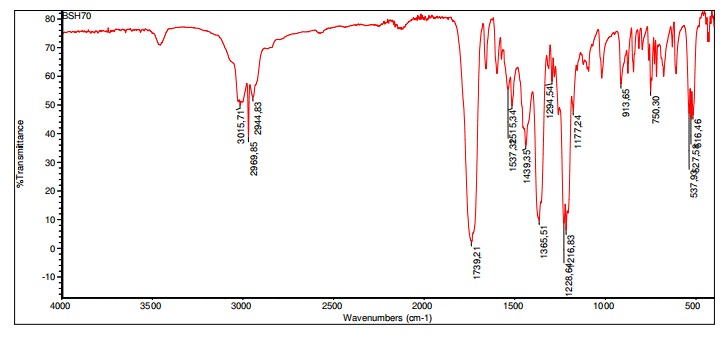


IR spectrum of N-(benzo[*d*]thiazol-2-yl)-4-methoxybenzamide (1)

Mass spectrum of N-(benzo[*d*]thiazol-2-yl)-4-methoxybenzamide (1)

Chromatogram of N-(benzo[*d*]thiazol-2-yl)-4-methoxybenzamide (1)

^1^H NMR spectrum of N-(benzo[*d*]thiazol-2-yl)-4-fluorobenzamide (2)

^13^C NMR spectrum of N-(benzo[*d*]thiazol-2-yl)-4-fluorobenzamide (2)


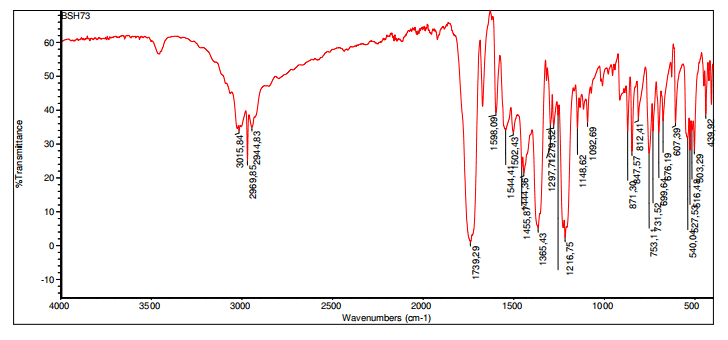


IR spectrum of N-(benzo[*d*]thiazol-2-yl)-4-fluorobenzamide (2)

Mass spectrum of N-(benzo[*d*]thiazol-2-yl)-4-fluorobenzamide (2)

Chromatogram of N-(benzo[*d*]thiazol-2-yl)-4-fluorobenzamide (2)

^1^H NMR spectrum of N-(benzo[*d*]thiazol-2-yl) thiophene-2-carboxamide (3)

^13^C NMR spectrum of N-(benzo[*d*]thiazol-2-yl) thiophene-2-carboxamide (3)


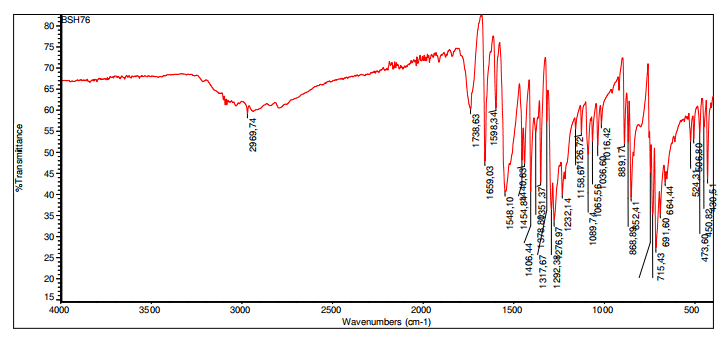


IR spectrum of N-(benzo[*d*]thiazol-2-yl) thiophene-2-carboxamide (3)

Mass spectrum of N-(benzo[*d*]thiazol-2-yl) thiophene-2-carboxamide (3)

Chromatogram of N-(benzo[*d*]thiazol-2-yl) thiophene-2-carboxamide (3)

^1^H NMR spectrum of N-(benzo[*d*]thiazol-2-yl) furan-2-carboxamide (4)

^13^C NMR spectrum of N-(benzo[*d*]thiazol-2-yl) furan-2-carboxamide (4)


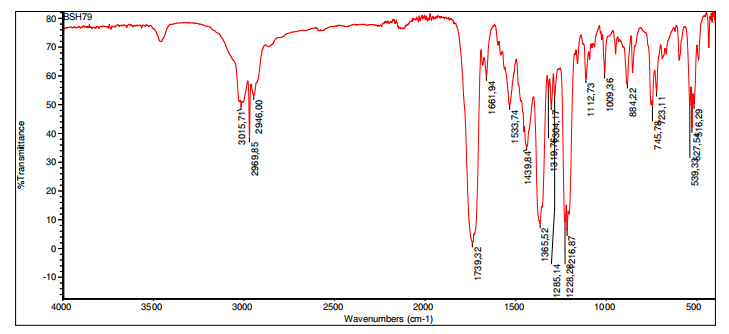


IR spectrum of N-(benzo[*d*]thiazol-2-yl) furan-2-carboxamide (4)

Mass spectrum of N-(benzo[*d*]thiazol-2-yl) furan-2-carboxamide (4)

Chromatogram of N-(benzo[*d*]thiazol-2-yl) furan-2-carboxamide (4)

^1^H NMR spectrum of N-(benzo[*d*]thiazol-2-yl)-4-bromobenzamide (5)

^13^C NMR spectrum of N-(benzo[*d*]thiazol-2-yl)-4-bromobenzamide (5)


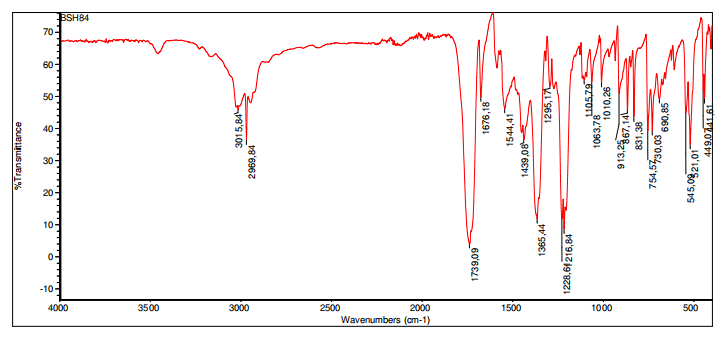


IR spectrum of N-(benzo[*d*]thiazol-2-yl)-4-bromobenzamide (5)

Mass spectrum of N-(benzo[*d*]thiazol-2-yl)-4-bromobenzamide (5)

Chromatogram of N-(benzo[*d*]thiazol-2-yl)-4-bromobenzamide (5)

^1^H NMR spectrum of N-(benzo[*d*]thiazol-2-yl)-4-(trifluoromethyl) benzamide (6)

^13^C NMR spectrum of N-(benzo[*d*]thiazol-2-yl)-4-(trifluoromethyl) benzamide (6)


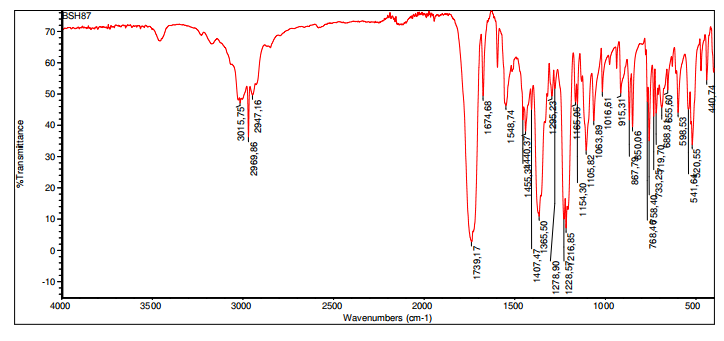


IR spectrum of N-(benzo[*d*]thiazol-2-yl)-4-(trifluoromethyl) benzamide (6)

Mass spectrum of N-(benzo[*d*]thiazol-2-yl)-4-(trifluoromethyl) benzamide (6)

Chromatogram of N-(benzo[*d*]thiazol-2-yl)-4-(trifluoromethyl) benzamide (6)

^1^H NMR spectrum of N-(benzo[*d*]thiazol-2-yl) benzamide (7)

^13^C NMR spectrum of N-(benzo[*d*]thiazol-2-yl) benzamide (7)


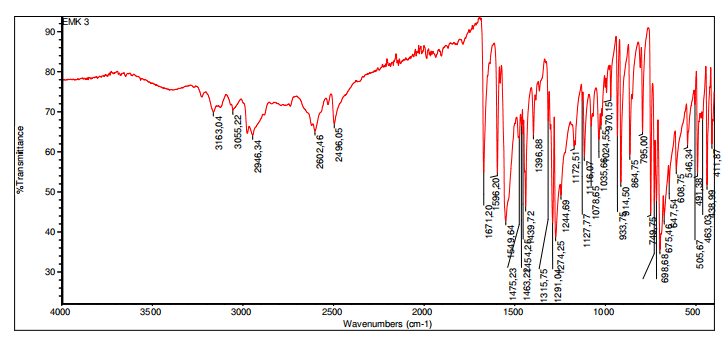


IR spectrum of N-(benzo[*d*]thiazol-2-yl) benzamide (7)

Mass spectrum of N-(benzo[*d*]thiazol-2-yl) benzamide (7)

Chromatogram of N-(benzo[*d*]thiazol-2-yl) benzamide (7)

^1^H NMR spectrum of N-(benzo[*d*]thiazol-2-yl)-4-methylbenzamide (8)

^13^C NMR spectrum of N-(benzo[*d*]thiazol-2-yl)-4-methylbenzamide (8)


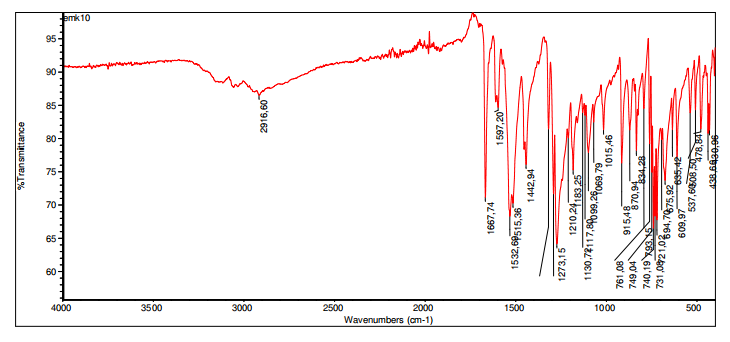


IR spectrum of N-(benzo[*d*]thiazol-2-yl)-4-methylbenzamide (8)

Mass spectrum of N-(benzo[*d*]thiazol-2-yl)-4-methylbenzamide (8)

Chromatogram of N-(benzo[*d*]thiazol-2-yl)-4-methylbenzamide (8)

^1^H NMR spectrum of N-(benzo[*d*]thiazol-2-yl)-4-chlorobenzamide (9)

^13^C NMR spectrum of N-(benzo[*d*]thiazol-2-yl)-4-chlorobenzamide (9)


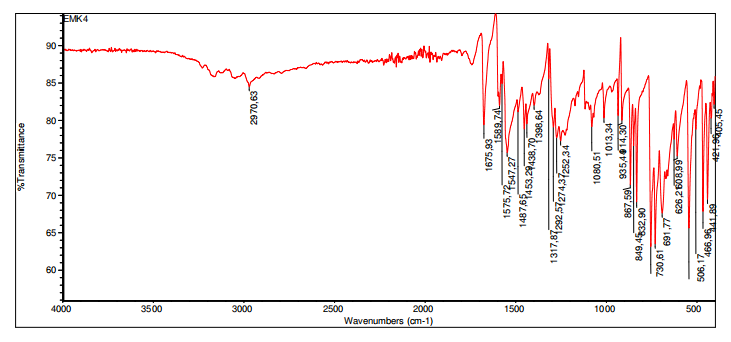


IR spectrum of N-(benzo[*d*]thiazol-2-yl)-4-chlorobenzamide (9)

Mass spectrum of N-(benzo[*d*]thiazol-2-yl)-4-chlorobenzamide (9)

Chromatogram of N-(benzo[*d*]thiazol-2-yl)-4-chlorobenzamide (9)

^1^H NMR spectrum of N-(benzo[*d*]thiazol-2-ylmethyl) benzamide (10)

^13^C NMR spectrum of N-(benzo[*d*]thiazol-2-ylmethyl) benzamide (10)


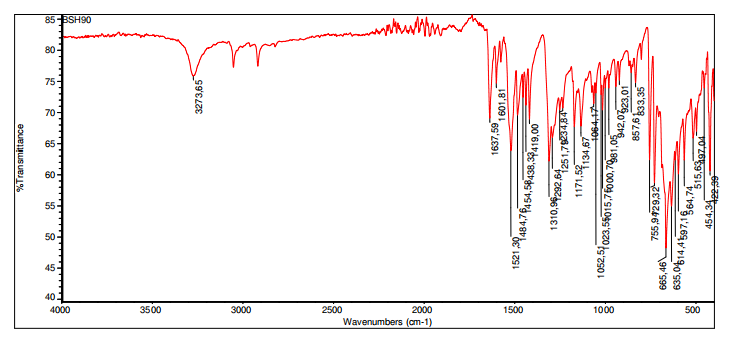


IR spectrum of N-(benzo[*d*]thiazol-2-ylmethyl) benzamide (10)

Mass spectrum of N-(benzo[*d*]thiazol-2-ylmethyl) benzamide (10)

Chromatogram of N-(benzo[*d*]thiazol-2-ylmethyl) benzamide (10)

^1^H NMR spectrum of N-(benzo[*d*]thiazol-2-ylmethyl)-4-chlorobenzamide (11)

^13^C NMR spectrum of N-(benzo[*d*]thiazol-2-ylmethyl)-4-chlorobenzamide (11)


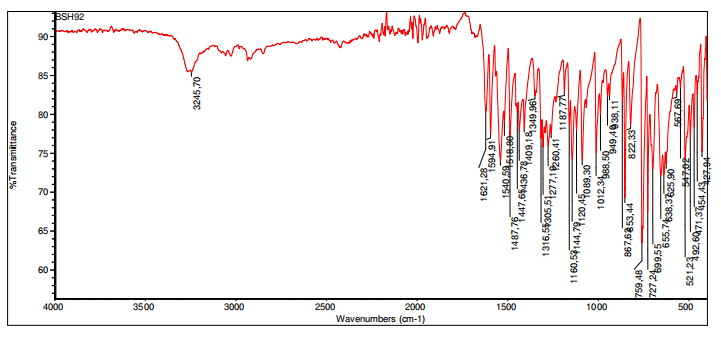


IR spectrum of N-(benzo[*d*]thiazol-2-ylmethyl)-4-chlorobenzamide (11)

Mass spectrum of N-(benzo[*d*]thiazol-2-ylmethyl)-4-chlorobenzamide (11)

Chromatogram of N-(benzo[*d*]thiazol-2-ylmethyl)-4-chlorobenzamide (11)

^1^H NMR spectrum of N-(benzo[*d*]thiazol-2-ylmethyl)-4-fluorobenzamide (12)

^13^C NMR spectrum of N-(benzo[*d*]thiazol-2-ylmethyl)-4-fluorobenzamide (12)


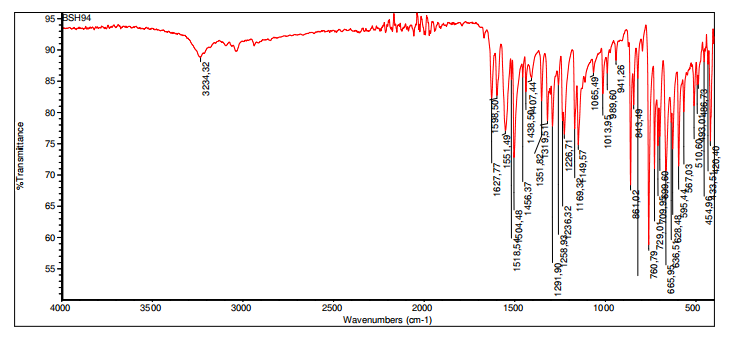


IR spectrum of N-(benzo[*d*]thiazol-2-ylmethyl)-4-fluorobenzamide (12)

Mass spectrum of N-(benzo[*d*]thiazol-2-ylmethyl)-4-fluorobenzamide (12)

Chromatogram of N-(benzo[*d*]thiazol-2-ylmethyl)-4-fluorobenzamide (12)

^1^H NMR spectrum of N-(benzo[*d*]thiazol-2-ylmethyl)-4-(trifluoromethyl) benzamide (13)

^13^C NMR spectrum of N-(benzo[*d*]thiazol-2-ylmethyl)-4-(trifluoromethyl) benzamide (13)


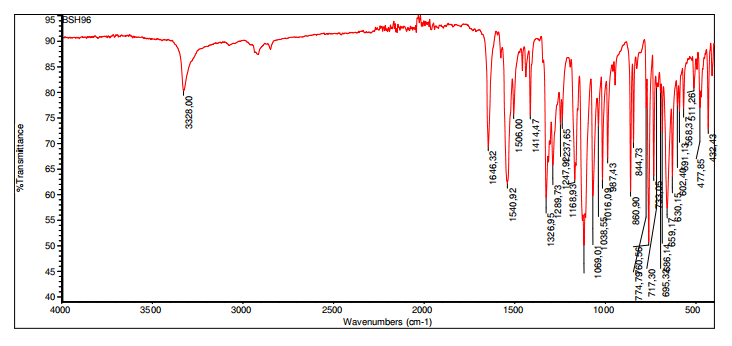


IR spectrum of N-(benzo[*d*]thiazol-2-ylmethyl)-4-(trifluoromethyl) benzamide (13)

Mass spectrum of N-(benzo[*d*]thiazol-2-ylmethyl)-4-(trifluoromethyl) benzamide (13)

Chromatogram of N-(benzo[*d*]thiazol-2-ylmethyl)-4-(trifluoromethyl) benzamide (13)

^1^H NMR spectrum of N-(benzo[*d*]thiazol-2-ylmethyl) thiophene-2-carboxamide (14)

^13^C NMR spectrum of N-(benzo[*d*]thiazol-2-ylmethyl) thiophene-2-carboxamide (14)


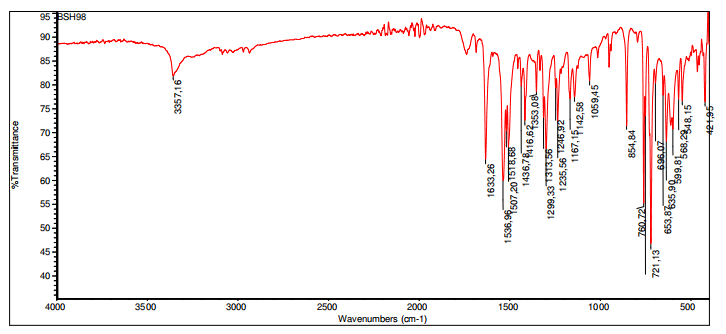


IR spectrum of N-(benzo[*d*]thiazol-2-ylmethyl) thiophene-2-carboxamide (14)

Mass spectrum of N-(benzo[*d*]thiazol-2-ylmethyl) thiophene-2-carboxamide (14)

Chromatogram of N-(benzo[*d*]thiazol-2-ylmethyl) thiophene-2-carboxamide (14)

^1^H NMR spectrum of N-(benzo[*d*]thiazol-2-ylmethyl) furan-2-carboxamide (15)

^13^C NMR spectrum of N-(benzo[*d*]thiazol-2-ylmethyl) furan-2-carboxamide (15)


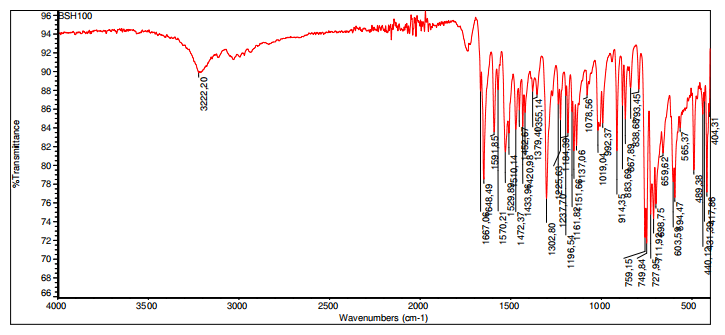


IR spectrum of N-(benzo[*d*]thiazol-2-ylmethyl) furan-2-carboxamide (15)

Mass spectrum of N-(benzo[*d*]thiazol-2-ylmethyl) furan-2-carboxamide (15)

Chromatogram of N-(benzo[*d*]thiazol-2-ylmethyl) furan-2-carboxamide (15)

^1^H NMR spectrum of N-(benzo[*d*]thiazol-2-ylmethyl)-4-bromobenzamide (16)

^13^C NMR spectrum of N-(benzo[*d*]thiazol-2-ylmethyl)-4-bromobenzamide (16)


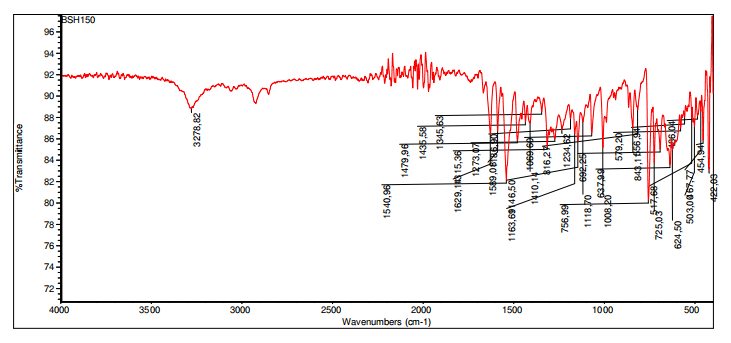


IR spectrum of N-(benzo[*d*]thiazol-2-ylmethyl)-4-bromobenzamide (16)

Mass spectrum of N-(benzo[*d*]thiazol-2-ylmethyl)-4-bromobenzamide (16)

Chromatogram of N-(benzo[*d*]thiazol-2-ylmethyl)-4-bromobenzamide (16)

^1^H NMR spectrum of N-(benzo[*d*]thiazol-2-ylmethyl)-4-methoxybenzamide (17)

^13^C NMR spectrum of N-(benzo[*d*]thiazol-2-ylmethyl)-4-methoxybenzamide (17)


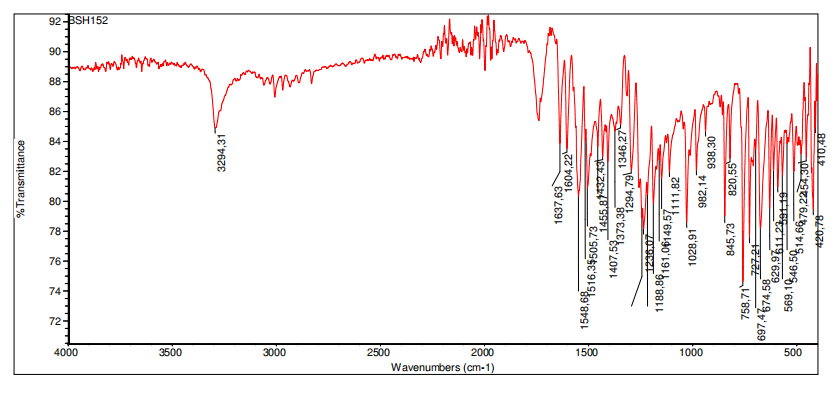


IR spectrum of N-(benzo[*d*]thiazol-2-ylmethyl)-4-methoxybenzamide (17)

Mass spectrum of N-(benzo[*d*]thiazol-2-ylmethyl)-4-methoxybenzamide (17)

Chromatogram of N-(benzo[*d*]thiazol-2-ylmethyl)-4-methoxybenzamide (17)

^1^H NMR spectrum of N-(benzo[*d*]thiazol-2-ylmethyl)-4-methylbenzamide (18)

^13^C NMR spectrum of N-(benzo[*d*]thiazol-2-ylmethyl)-4-methylbenzamide (18)


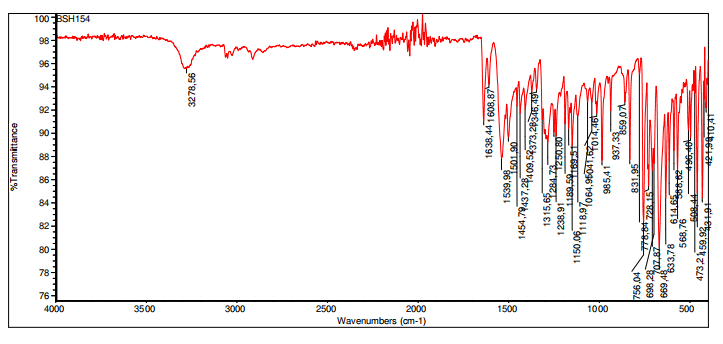


IR spectrum of N-(benzo[*d*]thiazol-2-ylmethyl)-4-methylbenzamide (18)

Mass spectrum of N-(benzo[*d*]thiazol-2-ylmethyl)-4-methylbenzamide (18)

Chromatogram of N-(benzo[*d*]thiazol-2-ylmethyl)-4-methylbenzamide (18)


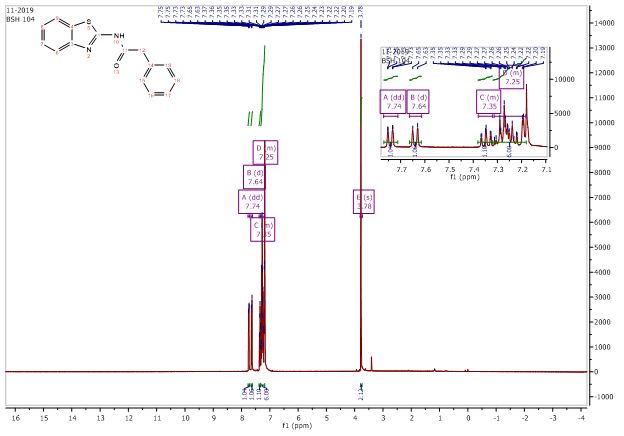


^1^H NMR spectrum of N-(benzo[*d*]thiazol-2-yl)-2-phenylacetamide (19)


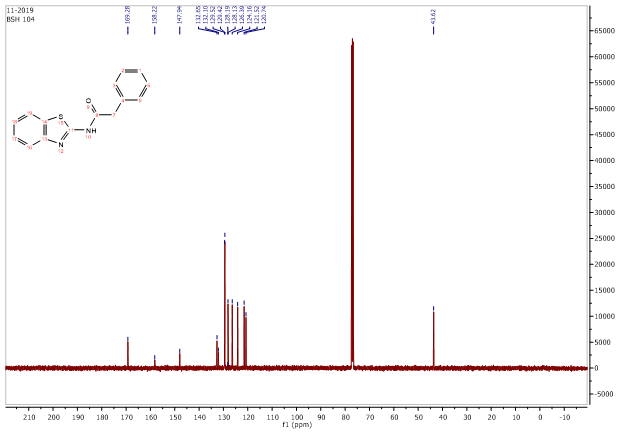


^13^C NMR spectrum of N-(benzo[*d*]thiazol-2-yl)-2-phenylacetamide (19)


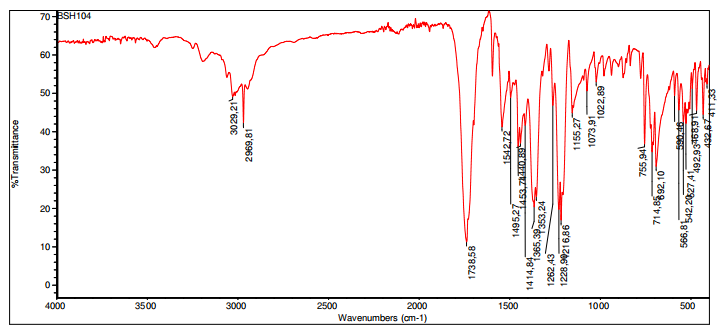


IR spectrum of N-(benzo[*d*]thiazol-2-yl)-2-phenylacetamide (19)

Mass spectrum of N-(benzo[*d*]thiazol-2-yl)-2-phenylacetamide (19)

Chromatogram of N-(benzo[*d*]thiazol-2-yl)-2-phenylacetamide (19)


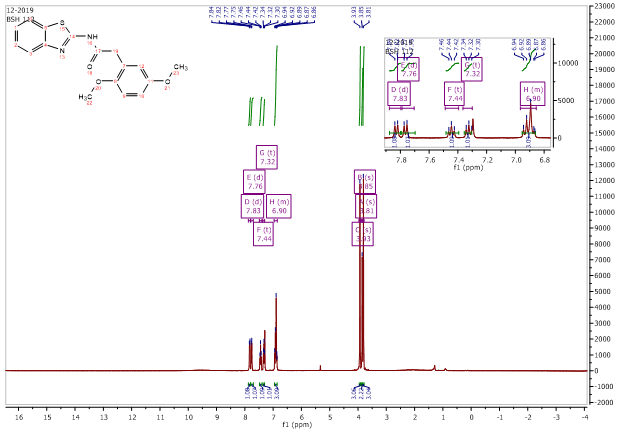


^1^H NMR spectrum of N-(benzo[*d*]thiazol-2-yl)-2-(2,5-dimethoxyphenyl) acetamide (20)


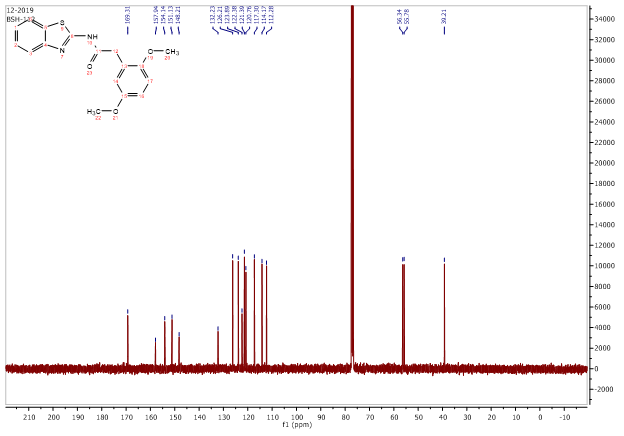


^13^C NMR spectrum of N-(benzo[*d*]thiazol-2-yl)-2-(2,5-dimethoxyphenyl) acetamide (20)


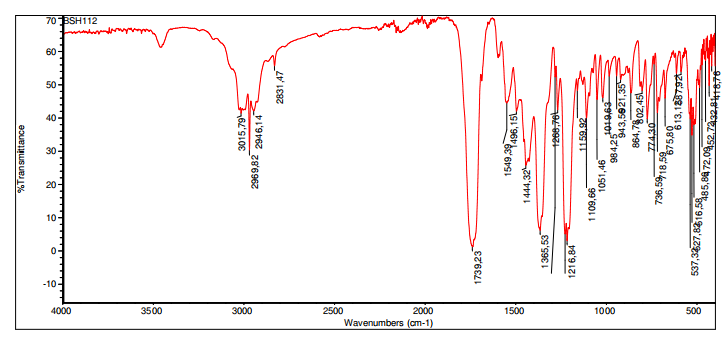


IR spectrum of N-(benzo[*d*]thiazol-2-yl)-2-(2,5-dimethoxyphenyl) acetamide (20)

Mass spectrum of N-(benzo[*d*]thiazol-2-yl)-2-(2,5-dimethoxyphenyl) acetamide (20)

Chromatogram of N-(benzo[*d*]thiazol-2-yl)-2-(2,5-dimethoxyphenyl) acetamide (20)


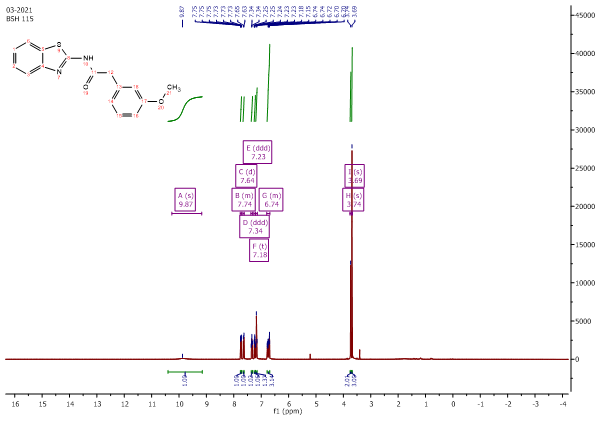


^1^H NMR spectrum of N-(benzo[*d*]thiazol-2-yl)-2-(3-methoxyphenyl) acetamide (21)


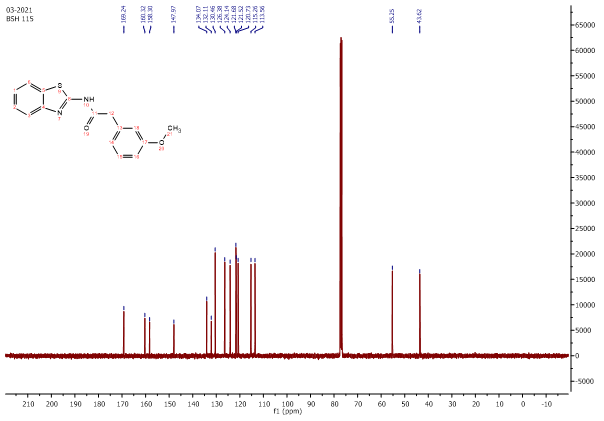


^13^C NMR spectrum of N-(benzo[*d*]thiazol-2-yl)-2-(3-methoxyphenyl) acetamide (21)


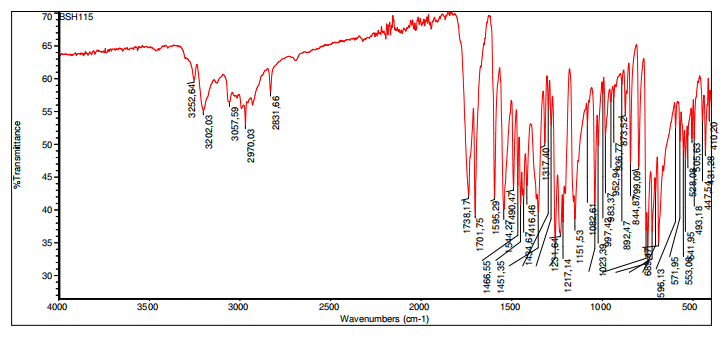


IR spectrum of N-(benzo[*d*]thiazol-2-yl)-2-(3-methoxyphenyl) acetamide (21)

Mass spectrum of N-(benzo[*d*]thiazol-2-yl)-2-(3-methoxyphenyl) acetamide (21)

Chromatogram of N-(benzo[*d*]thiazol-2-yl)-2-(3-methoxyphenyl) acetamide (21)

^1^H NMR spectrum of N-(benzo[*d*]thiazol-2-yl)-2-(4-fluorophenyl) acetamide (22)

^13^C NMR spectrum of N-(benzo[*d*]thiazol-2-yl)-2-(4-fluorophenyl) acetamide (22)


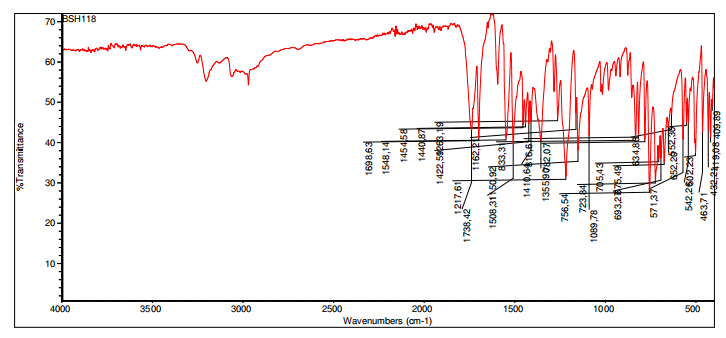


IR spectrum of N-(benzo[*d*]thiazol-2-yl)-2-(4-fluorophenyl) acetamide (22)

Mass spectrum of N-(benzo[*d*]thiazol-2-yl)-2-(4-fluorophenyl) acetamide (22)

Chromatogram of N-(benzo[*d*]thiazol-2-yl)-2-(4-fluorophenyl) acetamide (22)

^1^H NMR spectrum of N-(benzo[*d*]thiazol-2-yl)-2-(4-chlorophenyl) acetamide (23)

^13^C NMR spectrum of N-(benzo[*d*]thiazol-2-yl)-2-(4-chlorophenyl) acetamide (23)


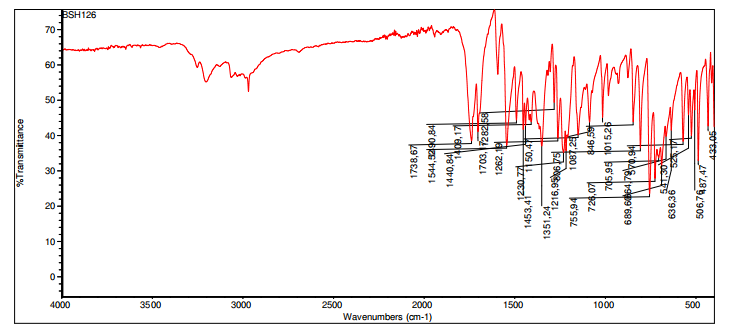


IR spectrum of N-(benzo[*d*]thiazol-2-yl)-2-(4-chlorophenyl) acetamide (23)

Mass spectrum of N-(benzo[*d*]thiazol-2-yl)-2-(4-chlorophenyl) acetamide (23)

Chromatogram of N-(benzo[*d*]thiazol-2-yl)-2-(4-chlorophenyl) acetamide (23)

^1^H NMR spectrum of N-(benzo[*d*]thiazol-2-yl)-2-(4-methoxyphenyl) acetamide (24)

^13^C NMR spectrum of N-(benzo[*d*]thiazol-2-yl)-2-(4-methoxyphenyl) acetamide (24)


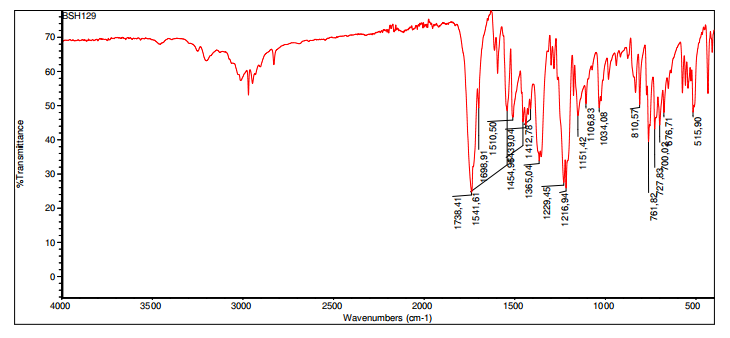


IR spectrum of N-(benzo[*d*]thiazol-2-yl)-2-(4-methoxyphenyl) acetamide (24)

Mass spectrum of N-(benzo[*d*]thiazol-2-yl)-2-(4-methoxyphenyl) acetamide (24)

Chromatogram of N-(benzo[*d*]thiazol-2-yl)-2-(4-methoxyphenyl) acetamide (24)

^1^H NMR spectrum of N-(benzo[*d*]thiazol-2-yl)-2-(thiophen-2-yl) acetamide (25)

^13^C NMR spectrum of N-(benzo[*d*]thiazol-2-yl)-2-(thiophen-2-yl) acetamide (25)


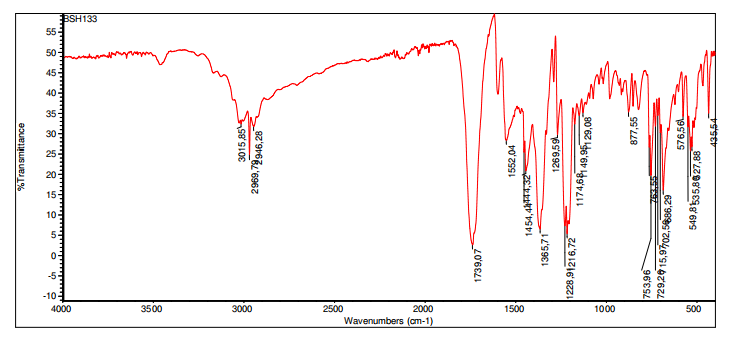


IR spectrum of N-(benzo[*d*]thiazol-2-yl)-2-(thiophen-2-yl) acetamide (25)

Mass spectrum of N-(benzo[*d*]thiazol-2-yl)-2-(thiophen-2-yl) acetamide (25)

Chromatogram of N-(benzo[*d*]thiazol-2-yl)-2-(thiophen-2-yl) acetamide (25)

^1^H NMR spectrum of N-(benzo[*d*]thiazol-2-ylmethyl)-2-(3-methoxyphenyl) acetamide (26)

^13^C NMR spectrum of N-(benzo[*d*]thiazol-2-ylmethyl)-2-(3-methoxyphenyl) acetamide (26)


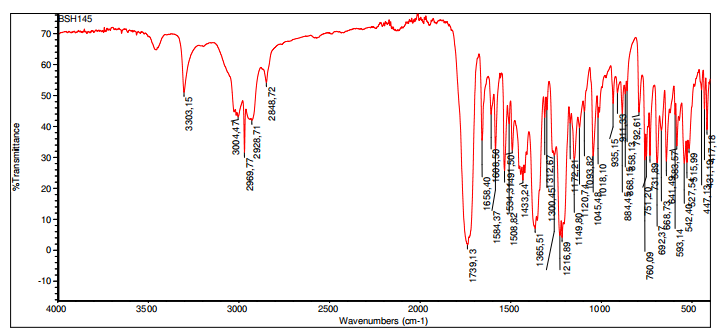


IR spectrum of N-(benzo[*d*]thiazol-2-ylmethyl)-2-(3-methoxyphenyl) acetamide (26)

Mass spectrum of N-(benzo[*d*]thiazol-2-ylmethyl)-2-(3-methoxyphenyl) acetamide (26)

Chromatogram of N-(benzo[*d*]thiazol-2-ylmethyl)-2-(3-methoxyphenyl) acetamide (26)

^1^H NMR spectrum of N-(benzo[*d*]thiazol-2-ylmethyl)-2-(4-chlorophenyl) acetamide (27)

^13^C NMR spectrum of N-(benzo[*d*]thiazol-2-ylmethyl)-2-(4-chlorophenyl) acetamide (27)


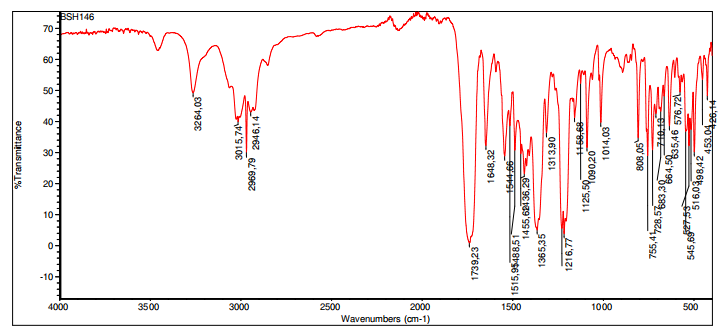


IR spectrum of N-(benzo[*d*]thiazol-2-ylmethyl)-2-(4-chlorophenyl) acetamide (27)

Mass spectrum of N-(benzo[*d*]thiazol-2-ylmethyl)-2-(4-chlorophenyl) acetamide (27)

Chromatogram of N-(benzo[*d*]thiazol-2-ylmethyl)-2-(4-chlorophenyl) acetamide (27)

^1^H NMR spectrum of N-(benzo[*d*]thiazol-2-ylmethyl)-2-(thiophen-2-yl) acetamide (28)

^13^C NMR spectrum of N-(benzo[*d*]thiazol-2-ylmethyl)-2-(thiophen-2-yl) acetamide (28)


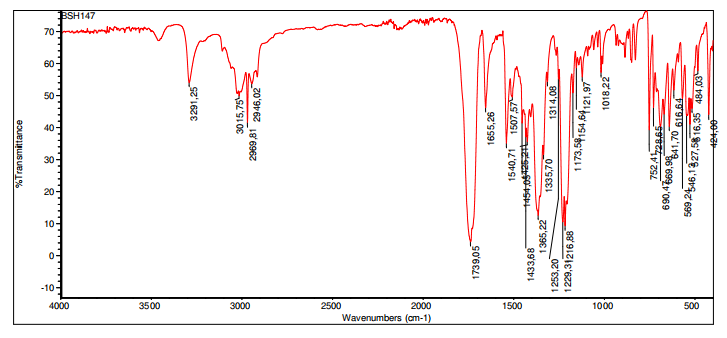


IR spectrum of N-(benzo[*d*]thiazol-2-ylmethyl)-2-(thiophen-2-yl) acetamide (28)

Mass spectrum of N-(benzo[*d*]thiazol-2-ylmethyl)-2-(thiophen-2-yl) acetamide (28)

Chromatogram of N-(benzo[*d*]thiazol-2-ylmethyl)-2-(thiophen-2-yl) acetamide (28)

**Table S1**. The crystal structures contain the known ligands (positive controls) and docking scores against 4DLI.

| **PDB ID** | **Resolution (Å)** | **Ligands** | **Docking Scores**  **kcal/mol** |
| --- | --- | --- | --- |
| 1ZZ2 | 2,00 | B11 | -7,45 |
| 2YIS | 2 | YIS | -6,48 |
| 3GCS | 2,1 | BAX(Sorafenib) | -6,337 |
| 3HP2 | 2,15 | P36 | -6,619 |
| 3IW7 | 2,4 | IPK | -6,344 |
| 3LFC | 2,8 | RL99(Z86) | -5,722 |
| 3NEW | 2,51 | 3NE | -8,51 |
| 3OBJ | 2,4 | BMU | -4,573 |
| 3UVP | 2,4 | O48 | -7,404 |
| 3ZS5 | 1,6 | SB2 | -8,23 |
| 3ZSG | 1,89 | TAK-175 (T75) | -7,576 |
| 3ZSH | 2,05 | SCIO-469 (469) | -5,038 |
| 3ZSI | 2,4 | VX-745 (52P) | -6,855 |
| 4DLJ | 2,6 | RL163 (6RG) | -7,836 |
| 4E6C | 2,34 | Perifosine (0O8) | -4,265 |
| 4EH2 | 2 | NP-F1 (0OK) | -7,565 |
| 4EH6 | 2,1 | NP-F5 (0ON) | -5,464 |
| 5N63 | 2,4 | 8OW | -7,973 |
| 5O8U | 2 | 9O5 | -8,427 |
| 6HWU | 2,3 | GE5 | -4,91 |
| 6HWV | 1,7 | GEW | -4,461 |

**Table S2**. The crystal structures contain the known ligands (positive controls) and docking scores against 1A9U.

| **PDB ID** | **Resolution (Å)** | **Ligands** | **Docking Scores**  **kcal/mol** |
| --- | --- | --- | --- |
| 1BL6 | 2,5 | SB216995 | -5,806 |
| 1DI9 | 2,6 | MSQ | -5,156 |
| 1M7Q | 2,4 | DQO | -4,829 |
| 1W84 | 2,2 | L12 | -5,228 |
| 2GFS | 1,75 | PQB | -5,46 |
| 2ZB1 | 2,5 | GK4 | -4,563 |
| 3E92 | 2 | G6A | -5,003 |
| 3FKN | 2 | FKN | -5,593 |
| 3FLS | 2,3 | FLS | -5,505 |
| 3FMJ | 2 | FMJ | -5,529 |
| 3FSF | 2,1 | FSS | -4,228 |
| 3GFE | 2,1 | P37 | -5,474 |
| 3HA8 | 2,48 | 5ZJ | -4,618 |
| 3ITZ | 2,25 | P66 | -4,85 |
| 3KQ7 | 1,8 | KQ7 | -4,94 |
| 3LHJ | 3,31 | LHJ | -5,252 |
| 3NWW | 2,09 | 3NW | -6,237 |
| 3RİN | 2,2 | I2O | -4,527 |
| 3U8W | 2,15 | 09J | -5,37 |
| 4AA4 | 2,3 | QC0 | -4,774 |
| 4kip | 2,27 | 1R9 | -4,568 |
| 4kiq | 2,5 | 1RA | -4,481 |
| 5omh | 2,5 | 9Y5 | -4,471 |
| 5tbe | 2,44 | 78L | -5,793 |
| 5wjj | 1,6 | AQY | -5,025 |
| 5xyx | 2,61 | FTZ | -5,454 |
| 5xyy | 1,7 | P0F | -5,462 |
| 6anl | 2 | T75 | -7,576 |
| 6m95 | 1,8 | J8S | -4,073 |
| 6m9l | 2,45 | J9G | -5,705 |
| 6sfi | 1,6 | LB5 | -5,031 |
| 6sfj | 1,95 | LBB | -4,981 |
| 6sfk | 1,8 | LB8 | -5,565 |
| 6sfo | 1,75 | LBE | -4,339 |
| 6y6v | 2,1 | OE5 | -4,026 |
| 6y6w | 1,9 | OE8 | -4,51 |
| 6y70 | 1,9 | OEB | -5,274 |

**
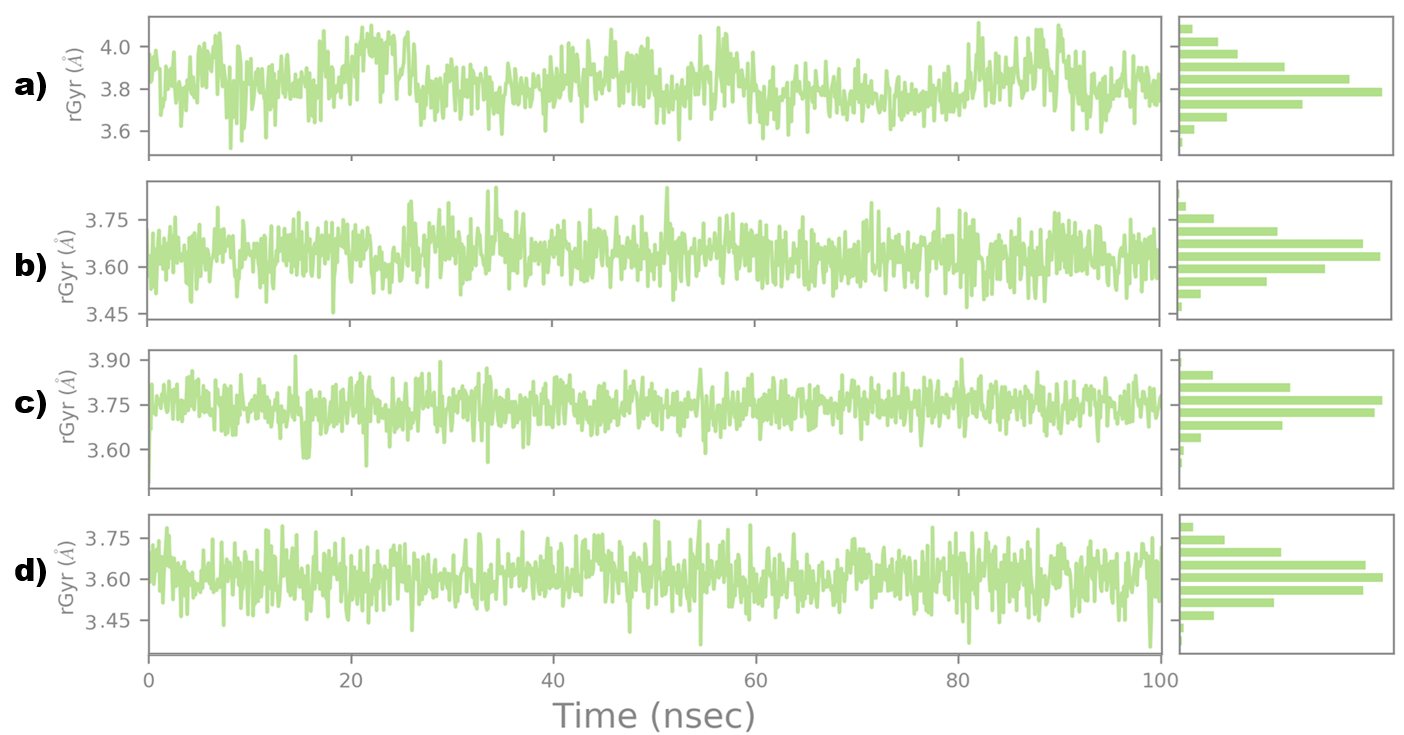
**

**Figure S1.** Radius of gyration (rGyr) analysis of the ligands in 4DLI and 1A9U during the 100 ns MD simulation. 19 in 1A9U **(a),** 19 in 4DLI **(b),** 15 in 1A9U **(c)**, 15 in 4DLI **(d)**

**
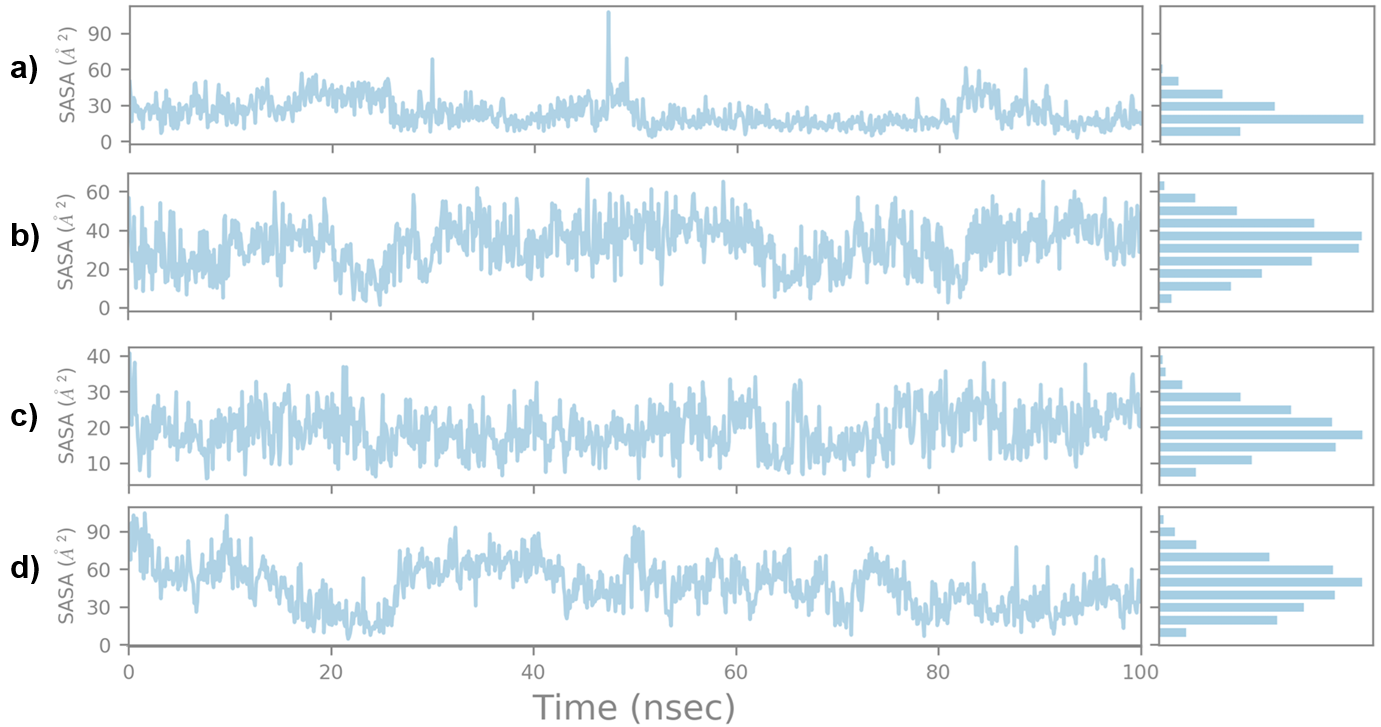
**

**Figure S2.** Solvent Accessible Surface Area (SASA) analysis of the ligands in 4DLI and 1A9U during the 100 ns MD simulation. 19 in 1A9U **(**a), 19 in 4DLI **(b)**, 15 in 1A9U **(c),** 15 in 4DLI **(d)**
